# Simulations of the Aqueous “Brown-Ring” Complex Reveal Fluctuations in Electronic Character

**DOI:** 10.1021/acs.inorgchem.3c02320

**Published:** 2023-10-02

**Authors:** Michael R. Coates, Ambar Banerjee, Michael Odelius

**Affiliations:** † Department of Physics, Stockholm University, AlbaNova University Center, SE-106 91 Stockholm, Sweden; ‡ Department of Physics and Astronomy, Uppsala University, Box 516, SE-751 20 Uppsala, Sweden

## Abstract

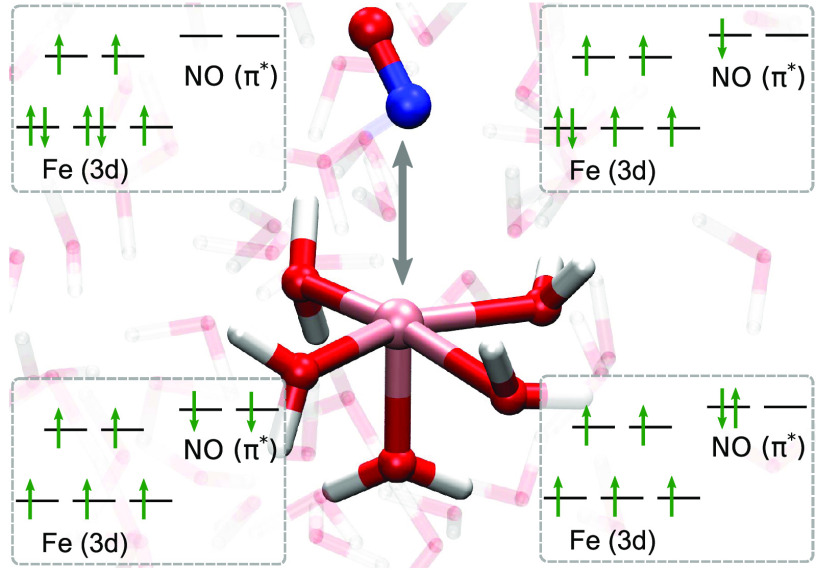

*Ab initio* molecular dynamics (AIMD) simulations of the aqueous [Fe(H_2_O)_5_(NO)]^2+^ “brown-ring” complex in different spin states, in combination with multiconfigurational quantum chemical calculations, show a structural dependence on the electronic character of the complex. Sampling in the quartet and sextet ground states show that the multiplicity is correlated with the Fe–N distance. This provides a motivation for a rigid Fe–N scan in the isolated “brown-ring” complex to investigate how the multiconfigurational wave function and the electron density change around the FeNO moiety. Our results show that subtle changes in the Fe–N distance produce a large response in the electronic configurations underlying the quartet wave function. However, while changes in spin density and potential energy are pronounced, variations in charge are negligible. These trends within the FeNO moiety are preserved in structural sampling of the AIMD simulations, despite distortions present in other degrees of freedom in the bulk solution.

## Abstract 2

The simplest aqueous iron-nitrosyl complex, the “brown-ring” complex has been studied by *ab intio* simulation and quantum chemistry calculations. Based on indications from the molecular dynamics simulations, the Fe−N distance is probed by multiconfigurational calculations. By means of structural sampling, structural correlations of the electronic density around the FeNO moiety are found to be present in the bulk liquid simulations.

## 1 Introduction

The nitrosyl ligand has attracted a lot of interest in the binding of NO in biological systems, in optical switches, and in precursor transition-metal complexes in a photoinduced nitric oxide-releasing mechanism. In biological systems, diiron enzymes^1^ detoxify NO anaerobically in cells by the activation and reduction of NO. Central to the study of iron-nitrosyl reactivity in biological systems is a detailed analysis of electronic structure, chemical bonding, and spin-state interactions. In heme proteins, it was essential to understand that a strong Fe(II)–NO σ-bond weakened the ligated Fe–N_His_ bond trans to the NO.^2^ In order to model specific properties of the FeNO moiety in proteins, nonheme and heme complexes prepared in {FeNO}^*n*^ (where *n* = 6, 7, 8)^3−6^ have been studied by both density functional theory (DFT) and multiconfigurational wave function approaches to elucidate the electronic configurations and the identity of high-spin/low-spin ground states. In optical switches, the metastable linkage isomerism of sodium nitroprusside (SNP) or [Fe(CN)_5_(NO)]^2–^ has been studied extensively by both experimental^7,8^ and theoretical^9−11^ approaches. The photoinduced isomerism that exchanges the Fe–NO configuration with the Fe–ON configuration has been largely studied theoretically by DFT calculations which have provided structural and orbital insights into the broader effect of linkage isomerism in transition-metal nitrosyls.^12^ To date, many nitric oxide-releasing precursor complexes have been prepared with chromium, manganese, and ruthenium,^13^ but recently, the use of iron-based complexes has been demonstrated.^14^ Iron-nitrosyl complexes typically have an absorption spectrum in the visible and ultraviolet (UV) regions, which, in the latter case, is damaging to biological systems. Due to the penetration depth of light into tissues and cells being dependent on wavelength, the targeted release of NO from a precursor complex can only be accomplished using near-infrared (NIR) light.^15^ As such, the local bonding environment around the FeNO moiety is tuned to produce NIR photorelease of NO, and hence a detailed theoretical understanding of the electronic configurations is necessary to understand the spin states involved in the initial release mechanism.^16^ In light of these studies, we investigate a well-known iron-nitrosyl solution in which the solvent influences the electronic structure of the FeNO moiety. We probe this rearrangement by means of a structural sampling coupled with a multiconfigurational treatment of the electronic wave function that allows us to investigate structural dependencies of electronic properties.

In the “brown-ring” reagent test from undergraduate analytical chemistry, the addition of an aqueous solution of Fe(II) to a solution containing nitric oxide forms a brown iron-nitrosyl complex at the liquid–liquid interface.^17−19^ The aqueous iron-nitrosyl complex, called the “brown-ring” complex [Fe(H_2_O)_5_(NO)]^2+^, belongs to a broad class of transition metal-nitrosyl (NO) complexes that are formed from the chemical exchange in a high-spin (*S* = 2) Fe(II) precursor complex replacing one of the coordinating ligands in the iron hexa-aqua complex with the nitrosyl molecule. Here, the open-shell doublet (*S* = 1/2) NO ligand binds to the iron center with the nitrogen end. Energetically close-lying and competing electronic configurations of the FeNO moiety result in a “non-innocence” of the coordinating NO ligand that presents challenges in the description of iron-nitrosyl complexes.^20^

**Figure 1:**
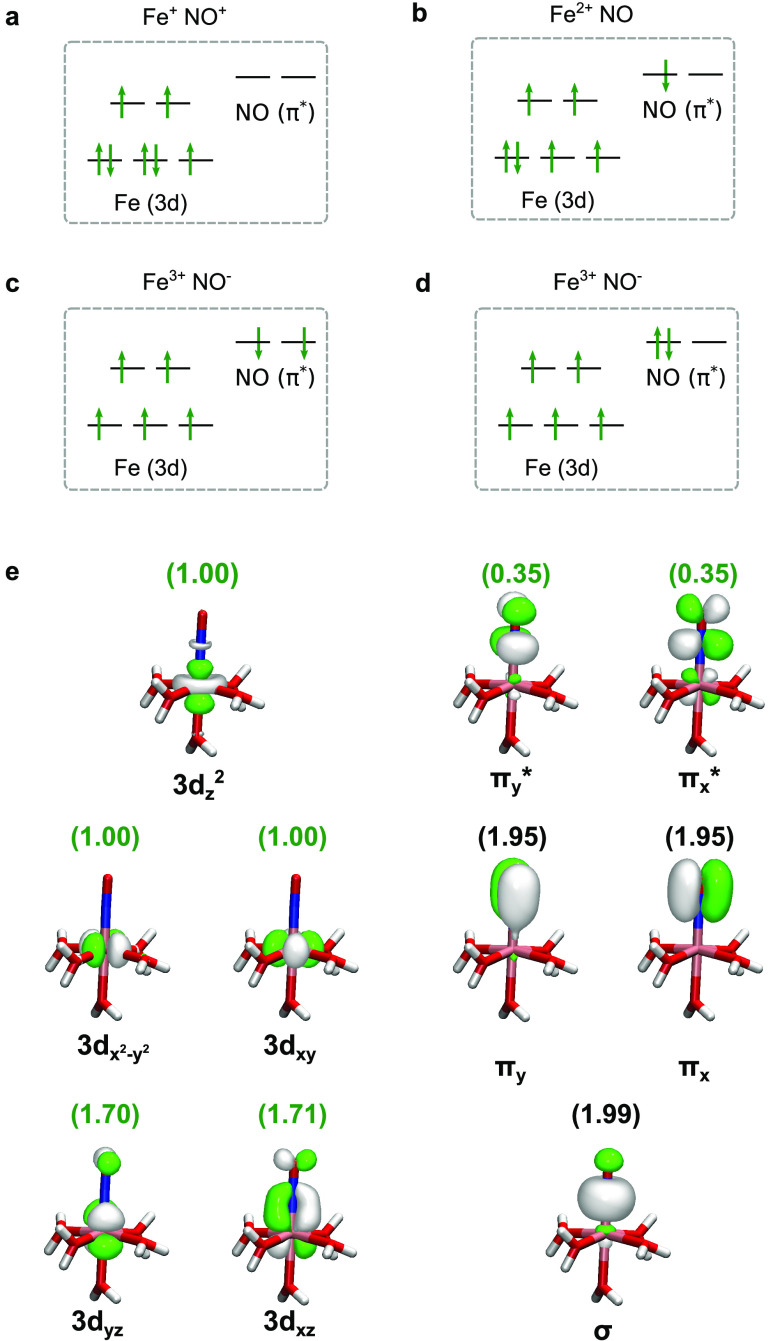
Different representations of the electronic configuration in the “brown-ring” complex. (a) Fe(I) metal and NO^+^ with a quartet (*S* = 3/2) 3d occupation. (b) Fe(II) metal and neutral NO with a quintet (*S* = 2) 3d occupation. (c) Fe(III) metal and triplet NO^–^ with a sextet (*S* = 5/2) 3d occupation. (d) Fe(III) metal and singlet NO^–^ with a sextet (*S* = 5/2) 3d occupation. (e) Molecular orbital (MO) diagram corresponding to the CASSCF (13e, 10o) active space used in the NEVPT2 calculations. The natural orbital occupations corresponding to the SA(1Q+1S)-CAS(13e,10o)/NEVPT2 CPCM(water) calculation are displayed above in parentheses, with the subset of orbitals in (a–c) shown in green.

The FeNO systems can be classified as {FeNO}^7^ according to the Enemark–Feltham notation,^21^ where the superscript denotes the total number of electrons in the FeNO moiety, that is in the “brown-ring” complex, with six electrons from the Fe 3d orbitals and one unpaired electron from the open-shell NO giving seven electrons and a total charge of 2+. Like other {FeNO}^7^ systems, the electronic structure, chemical bonding, and geometry of the “brown-ring” complex have been the subject of debate and interpretation.^20^ Alternative electronic configurations of [Fe(H_2_O)_5_(NO)]^2+^ that have been proposed are shown pictorially in Figure 1a–c, whereas Figure 1d is included for discussion in the context of the current study. It was first proposed in 1958 by Griffith et al.^22^ that the “brown-ring” complex was a Fe(I) species with all seven electrons residing on the metal center, giving a closed-shell NO^+^ cation shown in Figure 1a. This electronic configuration was assumed by Ogura et al.^23^ in their 1981 electrochemical study of Fe(II) with NO. The authors showed that the 450 nm band in the experimental UV spectrum was attributed to charge transfer within a Fe(I) metal center.

A kinetic and spectroscopic investigation in 2002 by Wanat et al.^24^ proposed that the structure was not a Fe(I) species but that the complex has a Fe(III) metal center as shown in Figure 1c. In this electronic configuration, two electrons are placed in the NO π* orbitals in a triplet (*S* = 1) NO^–^ anion that is antiferromagnetically coupled to the high-spin sextet (*S* = 5/2) Fe(III) metal center. This was followed by a theoretical investigation by Cheng et al.^25^ using DFT with the hybrid B3LYP functional. The authors proposed a revised electronic configuration, depicted in Figure 1b, with a quintet (*S* = 2) Fe(II) metal center antiferomagnetically coupled to a neutral doublet (*S* = 1/2) NO. In all three cases, the {FeNO}^7^ moiety preserves the total quartet (*S* = 3/2) multiplicity.

The establishment of a consistent bonding configuration in the “brown-ring” complex has been impaired by the debate of whether the FeNO moiety adopts an essentially linear (θ > 165°) or bent (θ < 140°) geometry. Gas-phase DFT calculations using the hybrid B3LYP functional by Cheng et al.^25^ and using the general gradient approximation (GGA) OLYP functional by Conradie et al.^26^ have shown a systematic preference for linearity. However, other {FeNO}^7^ complexes^26−28^ have shown a preference for a bent structure in the quartet (*S* = 3/2) spin multiplicity. Most recently, Monsch et al.^29^ have shown through crystal structure analysis using X-ray diffraction measurements that the complex appears as a slightly bent species with an Fe-N-O angle of 162.2° in the crystalline environment. The authors obtained structures from the crystallographic data and reoptimized implicitly solvated structures with the conductor-like polarizable continuum model (CPCM) parametrized for water using the GGA BP86 functional with the def2-TZVP basis set and obtained a bent structure with a Fe–N–O angle of 162.3°. The discrepancy of the bending angle was reflected in the prediction of the oxidation state, where the complete active space self-consistent field (CASSCF) description of the linear geometry predicted predominantly a Fe(II) metal center,^26^ as shown in Figure 1b, antiferromagnetically coupled to a neutral doublet (*S* = 1/2) NO species. The CASSCF description of the bent geometry predicted an essentially Fe(I) metal^29^ shown in Figure 1(a) bonded to a NO^+^ cation. Much like the Fe–N–O angle, the optimized Fe–N and N–O bond lengths have dependencies on and show variations with the choice of method, functional, basis set, and spin multiplicity. Monsch et al.^29^ showed a lengthened Fe–N bond attributed to back-bonding between the Fe 3d and NO π* orbitals, while Cheng et al.^25^ showed that the spin density of the NO was correlated with both the N–O bond length and the charge on the iron metal.

The discrepancies in the literature between DFT and wave function-based methods^20,22,24,25,29^ regarding structural information prompt a configurational sampling of the “brown-ring” complex in solution. Monsch et al.^29^ noted in the Supporting Information of their publication that the inclusion of explicit solvation in a complex with 10 H_2_O molecules results in a Fe–N–O bond angle of 148.0° with respect to the implicitly solvated structure with a Fe–N–O angle of 162.3°. To date, much of the interpretation of the structural information regarding the “brown-ring” complex has been through experimental probes like electron paramagnetic resonance (EPR) spectroscopy, Mössbauer, and flash photolysis spectroscopy in the study by Wanat et al.^24^ Conversely, most of the theoretical investigations supporting these experimental interpretations have exclusively used gas-phase calculations or implicit solvation models (PCM, COSMO).^25,26,30^ Recently, we reported^31^ on a liquid-phase *ab initio* molecular dynamics (AIMD) simulation in the quartet (*S* = 3/2) multiplicity using the BP86 GGA functional. The mechanistic details revealed that the previously established octahedral [Fe(H_2_O)_5_(NO)]^2+^ complex is present in the liquid along with a square-pyramidal [Fe(H_2_O)_4_(NO)]^2+^ species. Furthermore, based on multireference quantum chemical calculations used to reproduce the experimental ultraviolet–visible (UV–vis) spectrum, we proposed that the inclusion of the square-pyramidal species along with the classically established octahedral species results in an improved description of the three broad bands in the UV–vis spectrum.

The current study serves as an extension of the previous study by simulation of liquid-phase dynamics in the sextet (*S* = 5/2) multiplicity using the BP86 GGA functional and by simulations of liquid-phase dynamics in the quartet (*S* = 3/2) and sextet (*S* = 5/2) multiplicities using the BLYP GGA functional. Moreover, we combine the AIMD simulations^32^ with advanced multiconfigurational quantum chemistry using n-electron valence state perturbation theory (NEVPT2)^33,34^ to investigate fluctuations in nuclear and electronic degrees-of-freedom in the aforementioned metastable “brown-ring” complex in aqueous solution. From the AIMD simulations, the sampling of structures was performed to extract structural and electronic information about the aqueous [Fe(H_2_O)_5_(NO)]^2+^ and [Fe(H_2_O)_4_(NO)]^2+^ complexes at the DFT and NEVPT2 levels of theory. This forms the basis of a detailed discussion of the physical properties of the {FeNO}^7^ moiety.

## 2 Methods

The structure and dynamics of the aqueous “brown-ring” complex in the quartet and sextet states were investigated in AIMD simulations using the Car–Parrinello algorithm based on forces from DFT in the CPMD code version 4.3^32,35^ in a cubic cell (*a* = 21.6579 Å) at 300 K using the BP86^36^ and BLYP^37^ pure DFT functionals in the NVT ensemble. NEVPT2 calculations in ORCA version 4.2.0^38,39^ of electronic properties, sampled over the simulation trajectories, allowed for the determination of the multiconfigurational character of the electronic states. These results are compared to model calculations using the BP86 and BLYP pure functionals, the TPSSh^40^ hybrid functional, and previous NEVPT2 calculations.^31^ All electronic structure calculations complementing the AIMD simulations were performed by using ORCA.

### 2.1 *Ab Initio* Molecular Dynamics Simulations

Overall, four ∼40 ps long simulations were performed on the aqueous “brown-ring” complex, in a variation of quartet/sextet state and BP86/BLYP functional, and precise details are given below. The initialization, parameters, propagation, and analysis of the quartet BP86 AIMD trajectory are described in ref (31). All trajectories described hereafter are generated using the same simulation parameters as the quartet BP86 trajectory, and any deviations from that protocol are explicitly mentioned.

The sextet BP86 simulation was initiated from a configuration 3.7 ps into the quartet simulation, after which they were evolved separately. The sextet BP86 simulation was equilibrated from 3.7 ps until approximately 20 ps (see Figure S1), after which the remaining 42 ps was considered the production run of the simulation used for configurational sampling. By comparison, the quartet BP86 simulation had a production run of 45 ps, which was used for configurational sampling. The quartet and sextet BLYP simulations were performed with parameters identical to those of the BP86 simulation, with only the change in GGA functional type and associated pseudopotentials. The quartet BLYP simulation was initialized from the same initial condition as the quartet BP86 simulation described in ref (31) and then evolved separately. The quartet BLYP simulation was equilibrated for 20 ps after which the remaining 38.5 ps was considered the production run of the simulation. The sextet BLYP simulation was initiated from a configuration 4 ps into the quartet simulation, after which both trajectories were evolved separately. It evolved from 4 ps until 20 ps, after which the remaining 36 ps was considered the production run. The results of the BLYP simulations are summarized in Figures S2–S5 and discussed in the main text in comparison to the BP86 trajectories. The configurational sampling described in Section 2.2 is thus limited to the quartet and sextet BP86 production runs.

### 2.2 Quantum Chemistry Calculations

To enrich the analysis, the trajectories of quartet and sextet simulations were partitioned according to the number of water ligands around the iron into subsections with configurations of 5-coordinated [Fe(H_2_O)_5_(NO)]^2+^ and 4-coordinated [Fe(H_2_O)_4_(NO)]^2+^ “brown-ring” complexes. The *ad hoc* distinction between 4- and 5-coordinated species was based on an iron–water oxygen (Fe–O_w_) distance criteria of 3.0 Å taken from the oxygen number density minimum in the region between the first and second solvation shells. This allowed for an investigation into how steric interactions in the first solvation shell influence geometric and electronic degrees of freedom. The 5-coordinated [Fe(H_2_O)_5_(NO)]^2+^ and 4-coordinated [Fe(H_2_O)_4_(NO)]^2+^ complexes are denoted penta-aqua and tetra-aqua complexes hereafter.

Geometries were sampled in four distinct sets of configurations, obtained by taking 100 randomly selected trajectory snapshots from the subsections with penta-aqua and tetra-aqua coordinations in both the quartet and sextet trajectories. Each sampled configuration was pruned to exclude counterions and all solvent molecules, except for the five closest (according to the Fe–O_w_ distance) water molecules to the iron center. Hence, calculations for both the penta-aqua and tetra-aqua sampled geometries were performed on an iron-nitrosyl cluster with five water molecules. In each of the tetra-aqua clusters, the fifth closest water to the iron center is hydrogen bonded to one of the iron-coordinated water molecules as depicted in Figure 6 in our previous publication.^31^ For each sampled geometry, the atomic charges (*q*) and atomic spin densities (σ) obtained from the Mulliken population analysis, potential energy (*E*), and measures of multiconfigurational character based on the relative weight of the different configuration state functions (CFSs) contributing to the multiconfigurational wave function (, ···, and ···) were evaluated at the CASSCF level of theory. The potential energy (*E*) was further improved by the inclusion of dynamical correlation in the multiconfigurational wave function by calculating NEVPT2 energies for each sampled geometry. This was necessary to obtain accurate relative energies of the quartet and sextet states, offering an improvement over the CASSCF energies. We give the absolute energies (units hartree) in Table S1 and relative energies (units kcal mol^–1^) in Table S2 for the quartet, sextet, and doublet ground states at the TPSSh/def2-TZVP-optimized structure from ref (31). This provided a basis for the energetic exclusion of the doublet ground state at the NEVPT2 level of theory.

For each sampled configuration, the CASSCF calculation was state-averaged over the quartet and sextet ground states using the def2-TZVP basis set and with a def2-TZVP/C auxiliary basis set for integral transformations. The choice of active space in the construction of the CASSCF wave function is depicted in Figure 1e. In shorthand notation denoted as SA(1Q+1S)-CAS(13e,10o)/NEVPT2, it contains 13 electrons in 10 molecular orbitals. We use an alternative state averaging scheme to investigate the effect of state degeneracies through a state-averaged calculation with 10 quartet states and 9 sextet states, which we denote SA(10Q+9S)-CAS(13e,10o)/NEVPT2. The active space in the CASSCF and subsequent NEVPT2 calculations was chosen to include the orbitals coming from iron (3d_*xy*_, 3d_*yz*_, 3d_*xz*_, 3d_x^2^–*y*^2^_, 3d_z^2^_) and from the nitrosyl (σ, π_*x*_, π_*y*_, π_x_^*^, π_y_^*^). Solvent effects were treated with an implicit solvation model within the conductor-like polarizable continuum model (CPCM)^41^ and taking water as the solvent. Optimizations of these complexes in the quartet and sextet states were done using the BP86 and BLYP pure functionals to compare directly to the AIMD simulations and using the TPSSh hybrid functional to compare to the structures described in ref (31). The details of the Fe–N distances, N–O distances, and Fe–N–O angles for each of these structures are given in Table S3, while the associated Cartesian coordinates with absolute DFT energies are shown immediately below Table S3. Lastly, we complement the analysis done in ref (31) to establish the energetic argument for the presence of the penta-aqua and tetra-aqua equilibrium by calculating these complexes with the TPSSh, BP86, and BLYP functionals. The absolute and relative energies of the optimized complexes with each functional are given in Table S4.

## 3 Results and Discussion

### 3.1 AIMD Simulation of [Fe(H_2_O)_5_(NO)]^2+^ in Water

To assess the structural fluctuations of the “brown-ring” complex in an aqueous solution, AIMD simulations in the quartet and sextet electronic ground states were performed. In our previous study,^31^ the same quartet simulation was analyzed in detail and the analysis revealed the existence of penta-aqua and tetra-aqua complexes formed by the following exchange reaction in the first solvation shell
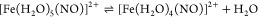
1This fluctuation between two hydration structures was characterized by constructing radial distribution functions (g(r)) for the five closest water molecules to the iron center. The results indicated a bimodal behavior of the fifth water molecule radial distribution function, showing the loss of axial water to the bulk solution. Radial distribution functions for the Fe–O_w_ distances showed that the equidistant point between the first and second solvation shell was approximately 3.0 Å (see Figure S6 for BP86 RDFs and Figure S7 for the BLYP RDFs). In the present study, the same analysis was applied to both the quartet and sextet simulations, therefore reproducing the results from the previous study involving the quartet simulation and extending the analysis to the sextet simulation. In doing so, we find that the bimodal distribution of the fifth closest water is present in both multiplicities. Although we cannot evaluate the statistics of the water-exchange process due to the limited number of exchange events in the short simulations, we consider the simulations sufficient to analyze structural and electronic fluctuations based on the sampling of structures. In Section S2.7 in the Supporting Information, we provide a detailed discussion about the validity of the AIMD simulations and the existence of the water-exchange process.

**Figure 2:**
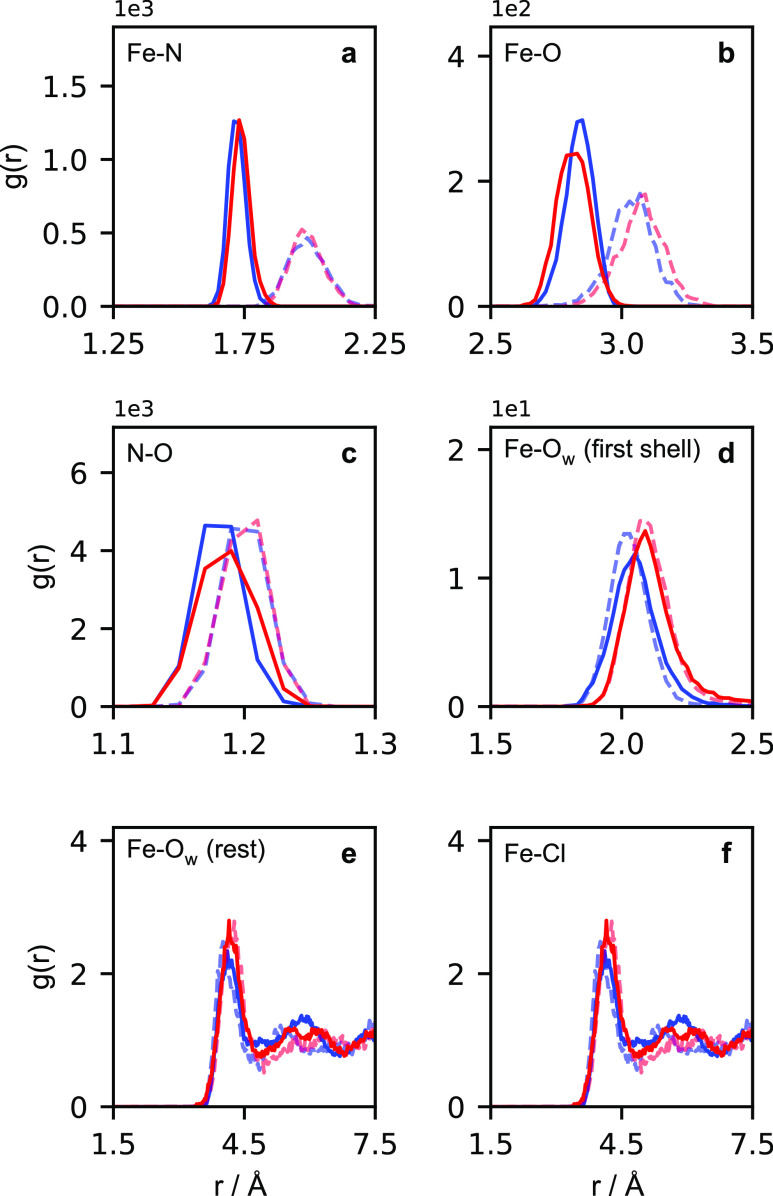
Radial distribution functions g(r) sampled from the AIMD trajectories of quartet and sextet simulations for distances between different pairs of atoms. (a) Fe–N, (b) Fe–O, (c) N–O, (d) first Fe–O_w_ solvation shell, (e) higher-order Fe–O_w_ solvation shells, and (f) Fe–Cl. The quartet simulations are represented as solid lines, while the sextet simulations are represented by opaque dashed lines. In addition, the differentiation between the penta-aqua and tetra-aqua in both simulations is indicated by the red color for the penta-aqua coordination and by the blue color for the tetra-aqua coordination.

Because of the dynamical equilibrium between the penta-aqua and tetra-aqua complexes and the separation of the first and second solvation shells, each simulation was partitioned into two separate sets of configurations, respectively, defined by the penta-aqua (Fe–O_w_^fifth^ < 3.0 Å) and tetra-aqua (Fe–O_w_^fifth^ > 3.0 Å) distances. Hence, the partitioning of configurations with different coordination from the two simulations for different multiplicity results in four sets of configurations denoted penta-aqua quartet, tetra-aqua quartet, penta-aqua sextet, and tetra-aqua sextet. Based on these four sets of configurations, g(r) for the Fe–N, Fe–O, N–O, Fe–O_w_, and Fe–Cl distances were constructed as shown in Figure 2a–f, where the water oxygen (O_w_) is distinguished relative to the nitrosyl oxygen (O). From the sampling of g(r), we can identify distinct differences in the coordination around the “brown-ring” complex within each multiplicity and between the multiplicities. In Figure 2a, the g(r) of the quartet simulation shows a sharp peak with a maximum centered around a Fe–N bond length of 1.73 and 1.71 Å for the penta-aqua and tetra-aqua complexes, respectively.

By comparison, the corresponding g(r) of the sextet simulations shows a Fe–N peak at longer distances with the Fe–N bond peak maxima at 1.97 and 1.99 Å for the penta-aqua and tetra-aqua complexes, respectively. The corresponding Fe–O distances in Figure 2b show a similar trend to that of the Fe–N coordinate, that is, the larger the Fe–N distance, the larger the Fe–O distance. In the quartet simulation, the Fe–O peak maxima are centered at 2.83 and 2.85 Å for the penta-aqua and tetra-aqua complexes, respectively. While in the sextet simulation, the Fe–O peak maxima are roughly 0.24 Å longer than that of the quartet simulation with the Fe–O maxima being 3.09 and 3.07 Å for the penta-aqua and tetra-aqua complexes, respectively. Finally, it is noted that the N–O bond lengths in Figure 2c, also show changes with multiplicity, but only a weak dependence on water coordination, when compared to the Fe–O distances. Between the two multiplicities, an overlap of the distributions indicates that the change in multiplicity has a small effect. In Figure 2d, we see the effect of the change in coordination on the first solvation shell likely due to the release of steric hindrance. In both cases, the tetra-aqua coordination has a g(r) with a distribution shifted to shorter Fe–O_w_ bond lengths, indicating that the exchange of the fifth water to the second solvation shell shortens the remaining Fe–O_w_ bonds in the first solvation shell. In Figure 2e, we see the second and third solvation shells, with the second solvation shell being relatively constant between both multiplicities and changes in coordination. The changes in the third solvation shell in Figure 2e,f are presented with reference to the distribution of Fe–Cl g(r). We see that the penta-aqua quartet and penta-aqua sextet Fe–Cl g(r) have significant overlap; as a consequence, the third Fe–O_w_ solvation shells are similar. By contrast, the tetra-aqua quartet and tetra-aqua sextet Fe–Cl g(r) have different distributions and the corresponding third Fe–O_w_ solvation shell shows clear differences in the shapes of the distributions. The statistics of the Fe–Cl exchange process with water in the second solvation shell are therefore limited and the description of this part of configuration space is deemed to be qualitative.

**Figure 3:**
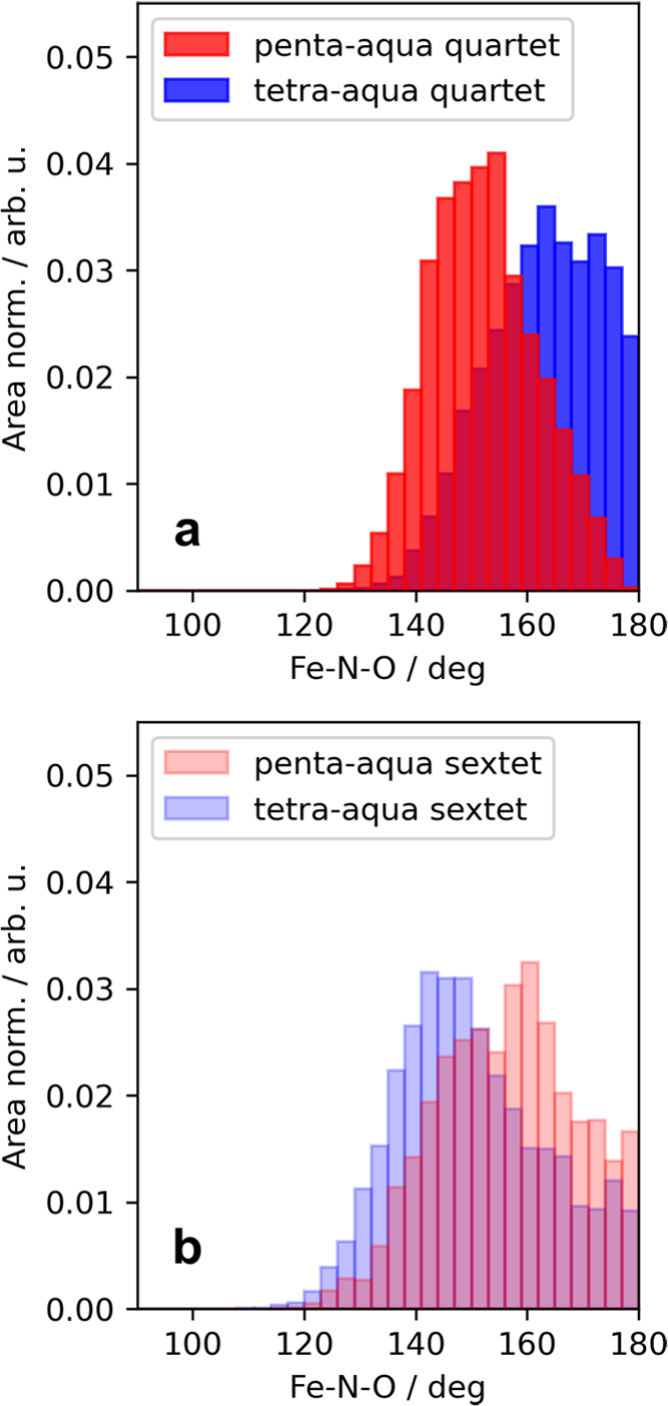
Distribution of angles from (a) quartet and (b) sextet AIMD trajectories. The angles sampled from the penta-aqua are colored in red, while those sampled from the tetra-aqua are colored in blue. All angle distributions are area normalized to compare peak intensities and all sampled angles are weighted by a 1/sin θ factor.

In light of the previous discussion in the literature, we additionally report changes in the Fe-N-O bending angle, θ_Fe–N–O_, shown in Figure 3.

The angle distributions are calculated by binning the θ_Fe–N–O_ values within each of the four sets of configurations and applying a bin weight of 1/sin(θ) to equate angles sampled from both the linear and bend regimes. This is done to account for the fact that the surface area on a sphere defined by the Fe-N-O bending angle tends to zero as the angle tends to linearity and tends to a maximum as the angle tends to 90°. We additionally area normalize the distributions. Using the same color scheme as before, the penta-aqua set is shown in red, the tetra-aqua set is shown in blue and the sextet sets are made opaque. We see in Figure 3a that the formation of the tetra-aqua quartet complex results in a shift from a bent FeNO moiety toward a more linear structure (note that previous studies refer that the θ_Fe–N–O_ > 165° is in the “linear” regime^26^). In contrast, in Figure 3b, the formation of the tetra-aqua sextet species results in a more bent FeNO moiety.

**Figure 4:**
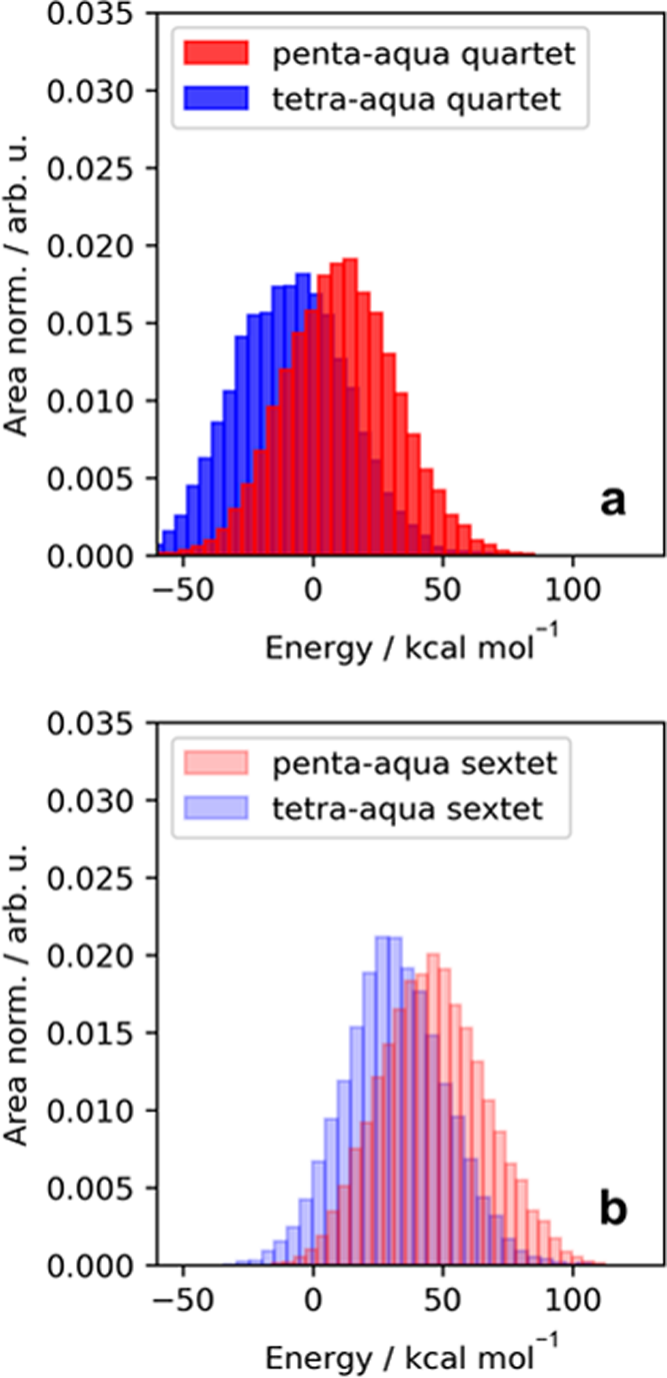
Distribution of potential (UKS) energies from (a) quartet and (b) sextet AIMD trajectories. The energies in the penta-aqua trajectory are colored in red while those in tetra-aqua are colored in blue. All energy distributions are area normalized to compare the peak intensities. The energy distributions are referenced relative to the average potential in the full quartet trajectory.

Finally, we also make a comparison of the distributions of the potential energies from the unrestricted Kohn–Sham (UKS) calculations underlying the AIMD simulations, taken from the four sets of configurations, with all potential energies represented as relative energies referenced to the average quartet energy. In Figure 4, we find that the tetra-aqua species is enthalpically favorable in both spin states, with a decrease in potential energy observed. We also find that the distributions of sextet potential energies have, on average, higher energy than the quartet. In Supporting Information Section S2.7 and Figure S8, we decompose the distributions into periodic DFT calculations and isolated cluster calculations to show how the bulk liquid enthalpically favors the exchange process. We additionally provide the same analysis for the BLYP functional in Figure S9. While the configurational sampling in Section 2.2 is limited to the BP86 set of configurations, we present the results of the BLYP simulations in Figures S2–S4. In Figure S2, in contrast to the results of the BP86 functional, the AIMD dynamics using the BLYP functional simulated in both the quartet and sextet multiplicities do not show any evidence of a dynamical equilibrium between a penta-aqua and tetra-aqua species during the production run. We refer the reader to Supporting Information Section S2.4, where we discuss the evidence of a dynamical equilibrium during the equilibration of the BLYP quartet AIMD trajectory in relation to the BP86 simulations. As a result, all AIMD dynamics results using the BLYP functional are presented in comparison to the aforementioned penta-aqua results from simulations with the BP86 functional. Figure S2a–f shows the g(r) for the same atom pairs in Figure 2a–f shows the Fe–N, Fe–O, N–O, Fe–O_w_, and Fe–Cl distances. With respect to the FeNO moiety, the same trends are observed for the two functionals. In Figure S2a, the penta-aqua quartet Fe–N g(r) shows a sharp peak with a maximum of around 1.77 Å, while the penta-aqua sextet Fe–N g(r) shows a broader distribution with a peak maximum at 2.03 Å, indicating a similar trend to the BP86 dynamics results. We observe the same trends in the N–O g(r), where the penta-aqua sextet set of configurations shows longer bond lengths than the corresponding penta-aqua quartet. A clear difference between the two functionals is the change in the Fe–Cl g(r), where in the dynamics simulated using the BLYP functional, we see closer Fe–Cl distances in the penta-aqua quartet set of configurations. However, we see no clear effect of this distance on the Fe–O_w_ solvation shells. We stress here that the statistics for the Fe–Cl distributions are limited based on the short AIMD simulations and the small fraction of deviations from the average Fe–Cl distances in the BLYP simulations. We report the changes in the Fe-N-O bending angle for the BLYP configurations in Figure S3. There are similarities between the distributions of the penta-aqua sextet configurations taken from the BLYP simulation in Figure S3b and from the BP86 simulation in Figure 3b, both of which show a broad distribution that contains both bent and linear Fe–N–O angles. Conversely, the penta-aqua quartet distribution of angles is shown in Figure S3a shows a preference for linear Fe–N–O angles, unlike the generally bent FeNO moiety obtained from the BP86 simulation in Figure 3a. We provide a discussion of the Fe–N–O angles using the BP86 and BLYP functionals in Section S2.7 and in Figure S10 in relation to the shifts in angular distributions in the AIMD simulations. Lastly, it should also be noted that in Figure S10, the potential energy surfaces related to the Fe–N–O angle are very flat and sensitive to the choice of the functional. Therefore, the analysis of the optimized structures in Table S3 and the associated Fe–N–O angles should be qualitative, as many energetically close-lying minima can be found.

**Figure 5:**
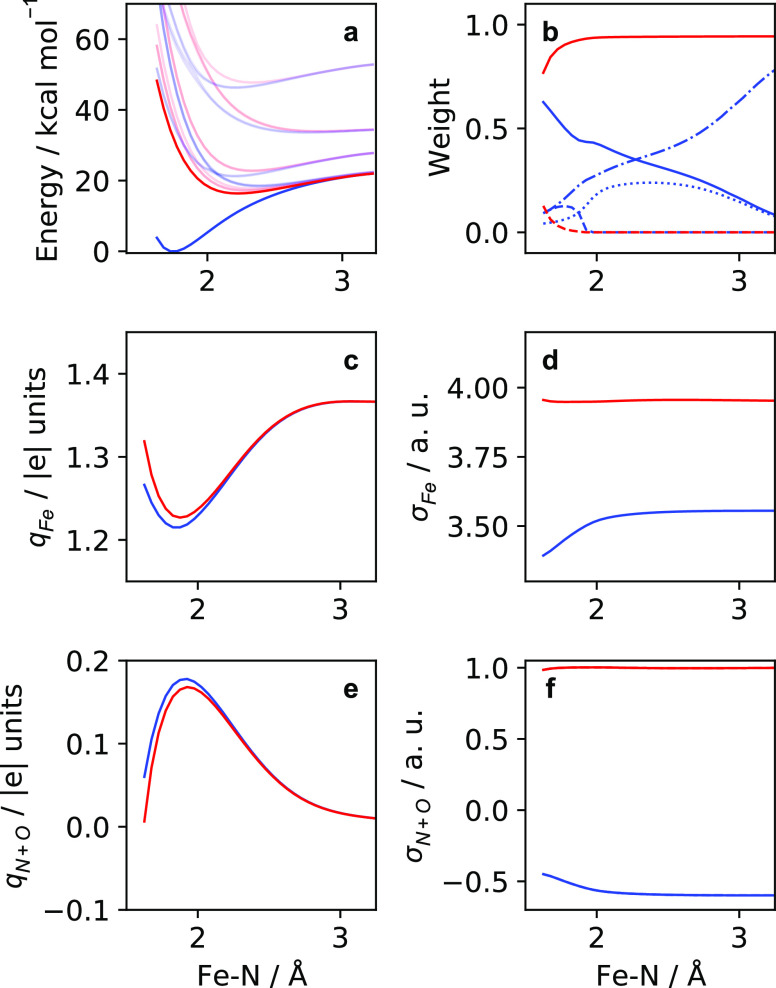
State properties for a rigid scan along the Fe–N scan calculated for the quartet (blue) and sextet (red) states at the SA(10Q+9S)-CAS(13e,10o)/NEVPT2 level of theory. (a) Potential energy curves of the electronic states. The ground state quartet and sextet states are shown bolded, with all excited states shown as opaque. (b) Weights of leading quartet CSFs defined in eq 2,  (solid blue),  (dot-dashed blue),  (dashed blue), and  (dotted blue) are shown. The weights of the two leading sextet CSFs defined in eq 3,  (solid red), and  (dashed red) are shown. (c, e) Mulliken atomic charges. (d, f) Mulliken atomic spin densities.

### 3.2 Fe–N Model Reaction

The analysis of g(r) in Figure 2 shows large changes in bonding within the FeNO moiety and a particular sensitivity to the change in multiplicity. In addition, the UKS AIMD potential energies show a sensitivity to both the change in multiplicity and the change in coordination in Figure 4. The binding of the NO moiety in its doublet state with a high spin iron in its quintet state couple produces the quartet and sextet spin states of the “brown-ring” complex. To understand the changes in structure, potential energy, and multiplicity, we probe the Fe–N distance by means of a scan. We examine this Fe–N distance to understand how the quartet and sextet states evolve, how their properties change, and how these states couple according to the Fe–N distance. The Fe–N distances were constructed by taking a rigid scan of the Fe–N bond at the interval 1.62–3.27 Å, starting with the penta-aqua quartet “brown-ring” complex optimized with implicit solvation at the TPSSh/def2-TZVP CPCM(water) level of theory. The choice of a rigid scan, as opposed to a relaxed scan in the quartet state, was done to reduce the degrees of freedom to only the Fe–N distance. In Figure S11, we considered the effects on the CASSCF, NEVPT2, and BP86 energies by comparing a rigid scan to a relaxed scan for geometries optimized using the SS(1Q)-CAS(13e,10o) CPCM(water) level of theory. We found that the CASSCF wave function predicts a dissociative potential not leading to coordination of the nitrosyl. Only when the dynamical correlation is added by NEVPT2 or by the exchange correlation in BP86, do we obtain the correct description of the Fe–N distances within the quartet minimum. In addition, we note that this points to the validity of the methods used here but also to the validity of the aforementioned BP86 AIMD simulations, which we discuss in Section S2.7. Furthermore, the exact mechanism of NO binding to the metal is not explored in the present study; instead, we focus on the explicit electronic structure changes in response to the Fe–N distance.

Along the rigid scan, the quartet and sextet potential energy surfaces are obtained using the aforementioned SA(1Q+1S)-CAS(13e,10o)/NEVPT2 CPCM(water) level of theory. To consider the effects of state degeneracies, we repeated the scan at the SA(10Q+9S)-CAS(13e,10o)/NEVPT2 CPCM(water) level of theory. The results of the SA(10Q+9S)-CAS(13e,10o)/NEVPT2 scan are summarized in Figure 5a, where the quartet (blue) and sextet (red) ground states are bold, while the additional excited states are shown as opaque. We find that the lowest sextet minimum is shallow and displaced approximately 0.4 Å from the quartet minimum and contains a number of close-lying states of the same multiplicity, while the quartet ground state is energetically well separated from all other quartet states. The remaining properties plotted in Figure 5b–f are taken from a SA(1Q+1S)-CAS(13e,10o)/NEVPT2 CPCM(water) calculation, which limits the state averaging to only two states. In addition, in Figure S12, we report the SA(1Q+1S+1D)-CAS(13e,10o)/NEVPT2 CPCM(water) energies and find that the inclusion of the doublet ground state along the Fe–N rigid scan distances indicates that the doublet ground state would not enrich the quartet–sextet interaction probed in this present study. We therefore restrict all further analysis to the quartet and sextet ground states.

We obtain the weight of the CSFs contributing to the wave function, defined in eq 2 for the quartet ground state and in eq 3 for the sextet ground state.
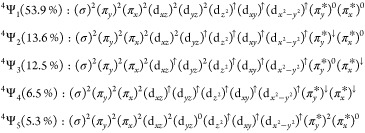
2
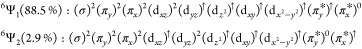
3

As previously reported,^20,29^ the quartet ground state is found to be multiconfigurational and composed of five leading electronic configurations defined in eq 2.  is the dominant CSF in the multiconfigurational wave function for the quartet ground state as depicted in Figure 1a. Here, all valence electrons in the FeNO moiety fill the iron d-orbitals, leaving the π_x_^*^/π_y_^*^ orbitals unoccupied. For  and , an electron is placed in the NO π_x_^*^/π_y_^*^ orbitals (see Figure 1b), giving a total spin of the quartet by antiferromagnetically coupling the electron to a spin-flipped electron in the iron d-orbitals. In the vicinity of the quartet minimum, these configurations hold a small weight relative to , where they account for roughly 25% of the wave function. We find two additional minor contributions to the wave function,  and , where  places two electrons in the NO π_x_^*^/π_y_^*^ orbitals in a triplet configuration that antiferromagnetically couple to electrons in the iron d-orbitals (see Figure 1c), while  corresponds to the doubly excited , which gives a singlet NO (see Figure 1d). We also report a corresponding doubly excited  CSF (relating to ), which has a weight of 1.34%. In Figure 5b, we plot the CSF weights for  CSF (solid blue),  CSF (dot-dashed blue),  CSF (dashed blue), and  CSF (dotted blue). We find that the sextet ground state is described almost entirely by a single  CSF in eq 3, taken to be the same CSF as  but with the π_y_^*^ electron having a spin flip. Similarly,  with a lower weight than  is taken to be the same CSF as , but with the π_x_^*^ electron having a spin flip. The  CSF is displayed as solid red and  CSF as dashed red in Figure 5b.

The  and  CSFs only have an apparent weight in the vicinity of the quartet minimum, where upon subsequent Fe–N elongation, the  CSF transfers all weight to the  CSF, resulting in a state described by a single electronic configuration. The character of the quartet becomes simplified outside of the quartet minimum as  tends to zero and the leading CSFs become mixed between , , and  in the vicinity of the sextet minimum. The  contribution tends to zero because the Fe–N bond lengthens and the overlap between one of the π* orbitals and an iron d-orbital becomes reduced. This preferentially occurs with one of the π* orbitals, since θ_Fe–N–O_ < 180° but even with a linear Fe–N–O angle, the presence of the axial water ligand trans to the nitrosyl causes breaking of the degeneracy of the π_*x*_/π_*y*_ and π_x_^*^/π_y_^*^ orbitals. In the vicinity of the sextet minimum, the doubly excited  CSF correlates with an increase in the singly excited  CSF and with the decrease of . In that minimum (at roughly 2.1 Å), the quartet wave function becomes even more multiconfigurational, reaching a maximum when the , , and  CSFs have a similar weight. The increase in the doubly excited  CSF preserves the overall neutral dissociation into a quintet Fe^2+^ and doublet neutral NO. The qualitative description of the , , and  CSFs shown pictorially in Figure 1 corresponded NO^+^, NO, and NO^–^ characters of the nitrosyl moiety, respectively. By excluding the  CSF, the wave function has a significant contribution from both CSFs corresponding to the NO^+^ and NO character of the nitrosyl moiety. The inclusion of the  CSF (which has a similar weight to the  CSF) balances out the wave function by including a CSF, which corresponds to an NO^–^ character of the nitrosyl moiety. Along the scan,  ultimately decays like the  CSF, until the neutral NO is dissociated. When the Fe–N bond is increased beyond the dissociation limit and degeneracy between the quartet and sextet ground states occurs, the quartet ground state becomes a single reference, dominated by . As previously stated, the leading CSF in the sextet multiplicity, , is the same electronic configuration as  but with the change of spin for the lone electron in the π_x_^*^/π_y_^*^ orbital, giving states that are coupled by the change in spin angular momentum. In a complementary analysis to Figure 5, we show the results of the wave function analysis in the relaxed scan in Figure S13. In Figure S13a, we find that while there exists a distortion of the quartet potential energy surface due to the bending of the Fe–N–O angle, the CSF weights in Figure S13c remain largely unchanged from the rigid scan.

In Figure 5c,d, we report the iron Mulliken atomic charges (*q*_Fe_) and the iron Mulliken atomic spin densities (σ_Fe_) as functions of the Fe–N distance. Furthermore, to compare the total FeNO moiety, the sum of the Mulliken atomic charges on N and O of the nitrosyl (*q*_N+O_) and the sum of the corresponding Mulliken atomic spin densities (σ_N+O_) are shown in Figure 5e,f, respectively. With respect to *q*_Fe_, the change between the quartet and sextet is effectively insignificant, where at long distances, these properties become equivalent. Moreover, in Figure 5e, the changes in *q*_N+O_ are inversely tied to the changes in *q*_Fe_, where the maximum value of q_N+O_ corresponds to the minimum value of *q*_Fe_. We note the approximate nature of Mulliken charges and instead reflect on the relative changes between the states. In a complementary analysis, we show the differences in charges along the rigid scan with the Mulliken, Voronoi deformation density (VDD), and Hirshfeld charge models in Figure S14. There we note that the observed behavior of the Mulliken charges is present in other charge models, while the magnitude of the charges changes significantly with each charge model.

In contrast to the Mulliken charges, we find significant changes in the Mulliken atomic spin densities in Figure 5d,f. In Figure 5d, σ_Fe_ is relatively constant along the Fe–N coordinate, corresponding to a value of approximately four. Recall that the measure of σ_Fe_ is derived from the number of unpaired electrons and hence for the sextet state described by the  CSF, which places four electrons in the Fe d-orbitals, we observe a value of four for σ_Fe_. Furthermore, with the  CSF, one electron is placed into a π* orbital shared between the nitrosyl and hence the value of one for σ_N+O_ is observed. We see a clear deviation from the expected value of three of σ_Fe_ for the quartet state and instead find values of σ_Fe_ between 3.4 and 3.55 between the quartet and sextet minima, tending to a value of approximately 3.6 at longer Fe–N distances. Since the quartet wave function is composed of the three leading CSFs of decreasing weight in eq 2, the spin density taken from these CSFs contains a description of the iron spin density with both three or four unpaired electrons in the d-orbitals. As a consequence, the highly multiconfigurational quartet ground state gives σ_Fe_ deviating from a value of three. Correspondingly, a σ_N+O_ value of zero would be expected for the quartet ground state, with three unpaired electrons residing in the iron d-orbitals. We see again the inverse relationship of σ_N+O_ with respect to σ_Fe_, where the description of σ_N+O_ is equally sensitive to the nature of unpaired spins in the nitrosyl ligand.

### 3.3 Configurational Sampling

**Figure 6:**
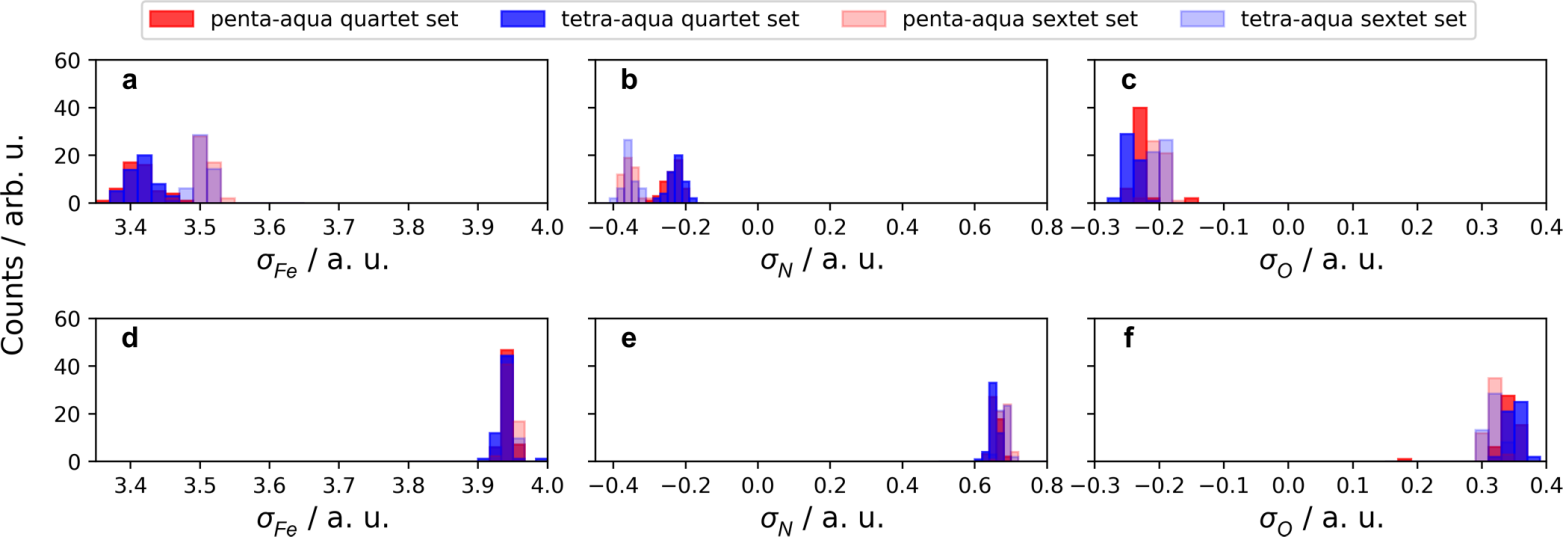
Atomic spin densities obtained from the SA(1Q+1S)-CAS(13e,10o) calculations. (a–c) Geometries sampled from the penta-aqua and tetra-aqua trajectories in the quartet multiplicity. (d–f) Geometries sampled from the penta-aqua and tetra-aqua trajectories in the sextet multiplicity. For all (a–f), the geometries sampled from the quartet sets of configurations are shown in bold, while the geometries sampled from the sextet sets of configurations are shown as opaque.

So far, we have considered a model Fe–N reaction by means of a scan that was proposed on the basis of the changes in the Fe–N g(r) in Section 3.1. Here, we report the results of the configurational sampling discussed in Section 2.2, presenting the distributions of the sampled properties, which have an implicit dependence on the changes in geometric information in Section 3.1. While both the Mulliken atomic charges and Mulliken atomic spin densities were presented in the Fe–N scan discussed in Section 3.2, we found the largest dependencies on Fe–N distances and changes in multiplicity to be with the Mulliken atomic spin densities. Figure 6a–f displays the sampled configurations in the same color scheme as the distributions of the θ_Fe–N–O_ in Figure 3.

As described in Section 3.1, we calculate all properties for all sampled geometries, and hence Figure 6a–c corresponds to the σ_Fe_, σ_N_, and σ_O_ Mulliken atomic spin densities for the quartet state, while Figure 6d–f corresponds to the σ_Fe_, σ_N_, and σ_O_ Mulliken atomic spin densities for the sextet state. We note, in all cases, the lack of sensitivity of the FeNO moiety to the change in water coordination in the first solvation shell, with all penta-aqua and tetra-aqua distributions overlapping. Instead, we find that geometries originating from both the quartet and sextet sets of configurations have a pronounced effect on the distributions of σ_Fe_ and σ_N_ for the quartet state in Figure 6a,b.

Knowing that the change in Fe–N distance is distinct from the Fe–N g(r) in Figure 2, we can think of the shifts in distributions to be similar to the changes in σ_Fe_ based on the Fe–N scan in Figure 5d. Similarly, the sampled σ_N_ values in Figure 6b indicate that the geometries sampled from the sextet show larger negative densities than the geometries sampled from the quartet. This is another indication of the manifestation of the Fe–N coordinate changes in the sampled properties.

**Figure 7:**
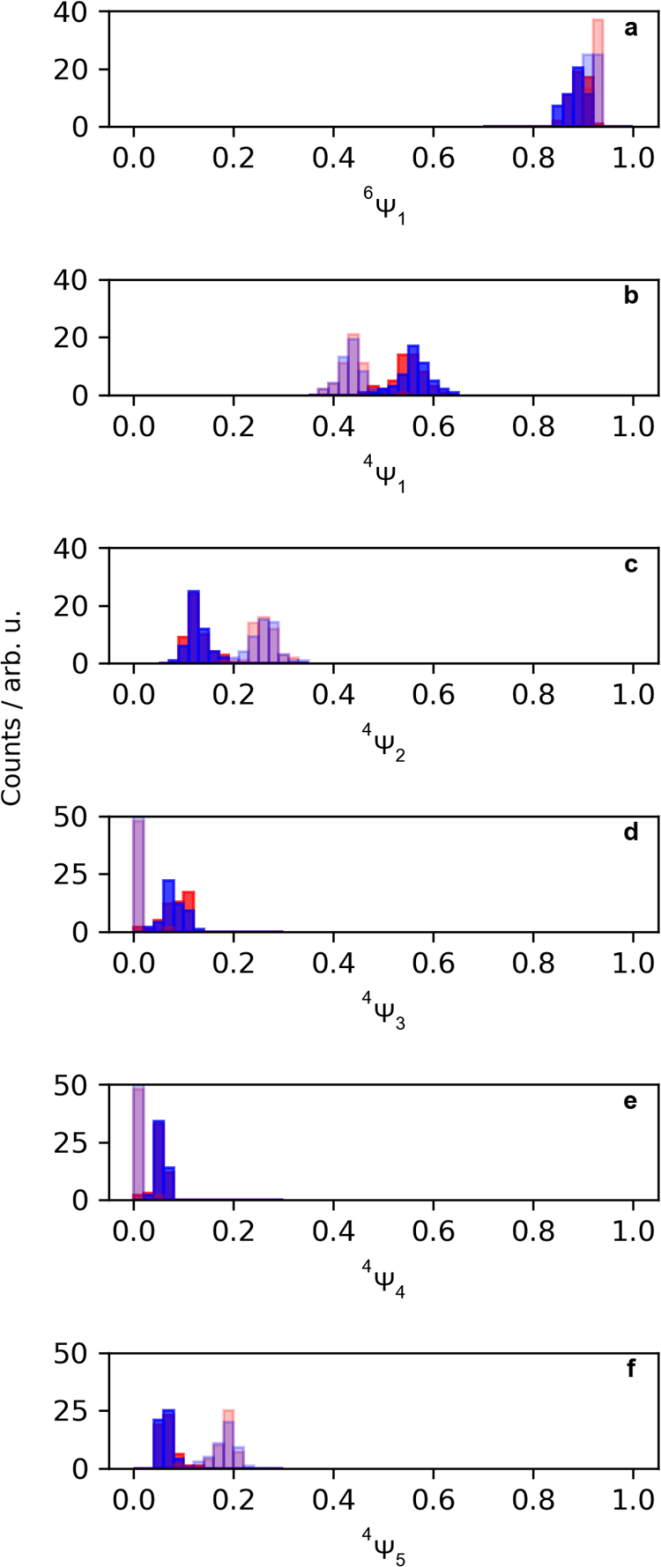
(a) Binned value of the weights  in eq 3 for all sampled geometries taken from the SA(1Q+1S)-CAS(13e,10o) calculations in the sextet multiplicity. (b–f) Binned values of weights , , , , and  in eq 2 for all sampled geometries taken from the SA(1Q+1S)-CAS(13e,10o) calculations in the quartet multiplicity.

For all sextet spin densities in Figure 6d–f, there is a lack of sensitivity to both the water coordination and all other possible distortions in geometries sampled from both electronic states. The spin densities in the scan in Figure 5d,f show that there was no significant change in σ_Fe_ and σ_N+O_. We find this to be present in the sampled data in Figure 6d–f, with the σ_Fe_ distribution centered around 3.9 and the sum of the σ_N_ and σ_O_ distributions giving values close to 0.9. Since the sampled σ_Fe_, σ_N_, and σ_O_ for the sextet state are constant in Figure 6d–f, but we see clear changes in the distributions of σ_Fe_, σ_N_, and σ_O_ for the quartet state in Figure 6a–c, we explore the characters of the wave function in the sampling of the quartet and sextet CSFs defined in eqs 2 and3. We show the sampled sextet CSF weight () in Figure 7a and the quartet CSF weights (, , , , and ) in Figure 7b–d.

It is clear from Figure 7a that the sextet state is almost exclusively described by a single reference wave function. We find this to be analogous to the  CSF in the Fe–N scan, which showed a particular insensitivity to the change in Fe–N distance. By contrast, we find there to be a dependence on the identities of the sampled geometries present in the sampled , , , and  CSF weights. Most notably, there is a decrease of  and increase of  and  for the geometries sampled from the sextet sets of configurations. With the changes in the Fe–N g(r) distributions in Figure 2, we anticipate that the shifts in , , and  distributions are reflective of the idealized shifts along the Fe–N scan in Figure 5b. We additionally find that all of the geometries sampled from the sextet sets of configurations have  and  CSF weights of zero, indicating that the geometric shifts transfer all of the weight to the two leading CSFs.

**Figure 8:**
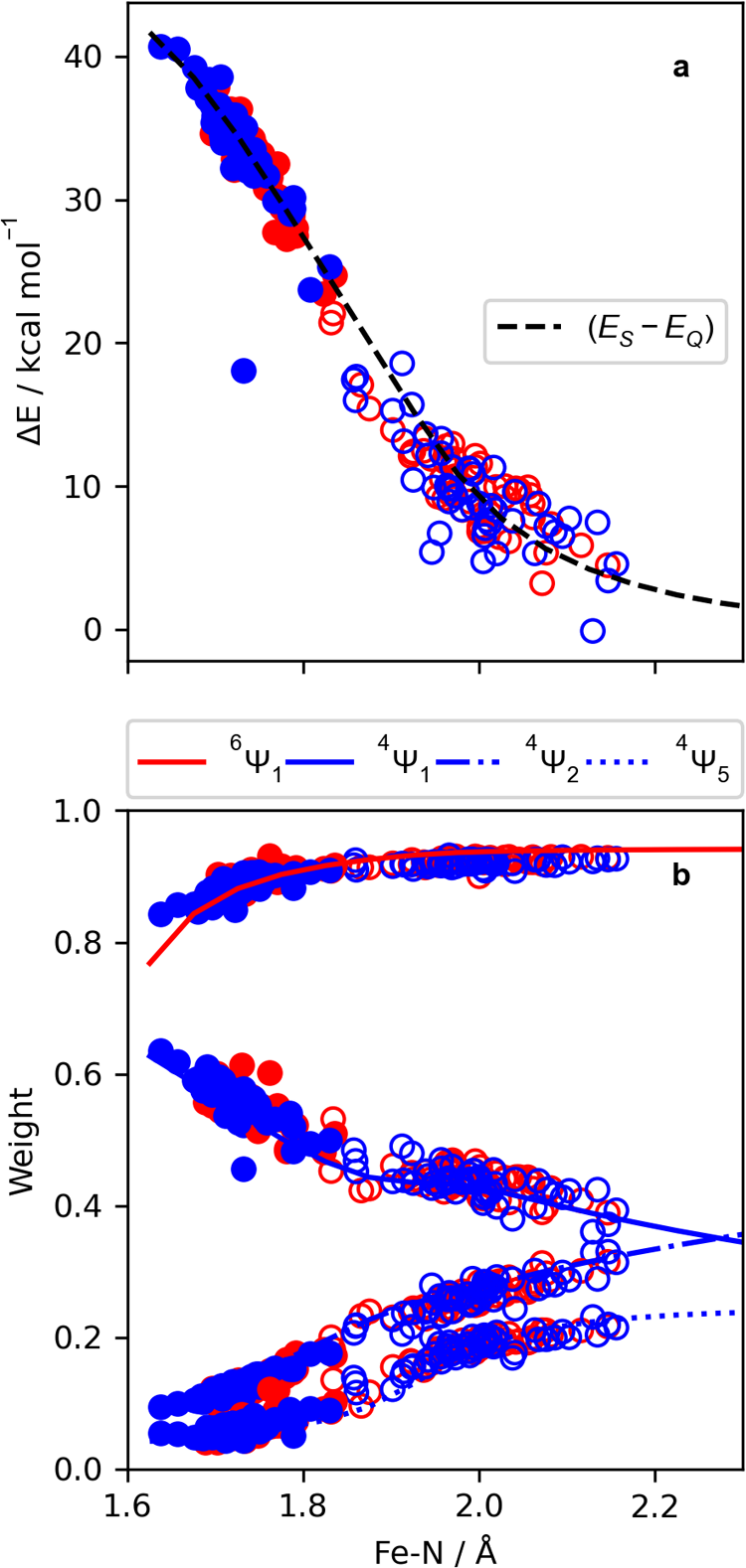
(a) Difference in NEVPT2 energies (sextet–quartet) for geometries sampled from the quartet and sextet trajectories. The penta-aqua sampled geometries are shown in red and the tetra-aqua sampled geometries are shown in blue. The geometries sampled from the quartet trajectory are shown as solid, while the geometries sampled from the sextet trajectory are shown to be hollow. The difference in energy taken from the model potentials in Figure 5a is shown by the dashed black line. (b) Weights of  (solid blue),  (dot-dashed blue),  (dotted blue), and  (solid red) CSFs taken from Figure 5b, which is plotted with the sampled data. The geometries sampled from the quartet trajectory are shown solid, while the geometries sampled from the sextet trajectory are shown to be hollow. The penta-aqua sampled geometries are shown in gray, while the tetra-aqua trajectories are shown in green. Unlike in (a), the measure of each electronic configuration corresponds only to the electronic configurations of the specified quartet or sextet state and not the state difference.

To see how *r*_Fe–N_ is represented in the AIMD trajectory data, we consider the effect of the sampled electronic properties with a change in bond length. By doing so, we project all of the sampled data on to the Fe–N scan defined in Section 3.2. The projection of the sampled NEVPT2 energies and CSF weights of the corresponding SA(1Q+1S)-CAS(13e,10o) wave function is shown in Figure 8.

In Figure 8a, the difference in energy between the sextet and quartet states calculated at the SA(1Q+1S)-CAS(13e,10o)/NEVPT2 level of theory is shown. This difference in energy is used to relate the sextet and quartet states while removing energetic shifts introduced by the structural variations in the surrounding liquid. Here, the Fe–N scan range is reduced to 1.6–2.3 Å, being above the limits of the Fe–N distances of the quartet and sextet simulations. For each sampled geometry, the difference in energy is calculated and projected onto the difference in energy of the sextet and quartet ground states in the Fe–N scan. We find an excellent agreement of the sampled geometries with the Fe–N scan, indicating a strong correlation of the Fe–N coordinate in the liquid simulation. In Figure 8a, we note the geometric distinction between the energies from geometries sampled from the quartet and sextet sets of configurations and also the lack of distinction between the solvation of the FeNO moiety. In Figure 8b, we display the sampled , , , and  CSF weights along the model Fe–N CSF weights from Figure 5b. Here, we again find excellent agreement, indicating the importance of the Fe–N distance in the liquid simulation. In Figure S15, we make a comparison to the rigid scan in Figure 8 by projecting the sampled energies and CSF weights on to the relaxed scan. We find there to be no substantial difference in the overall trends with respect to either model of the Fe–N distance.

**Figure 9:**
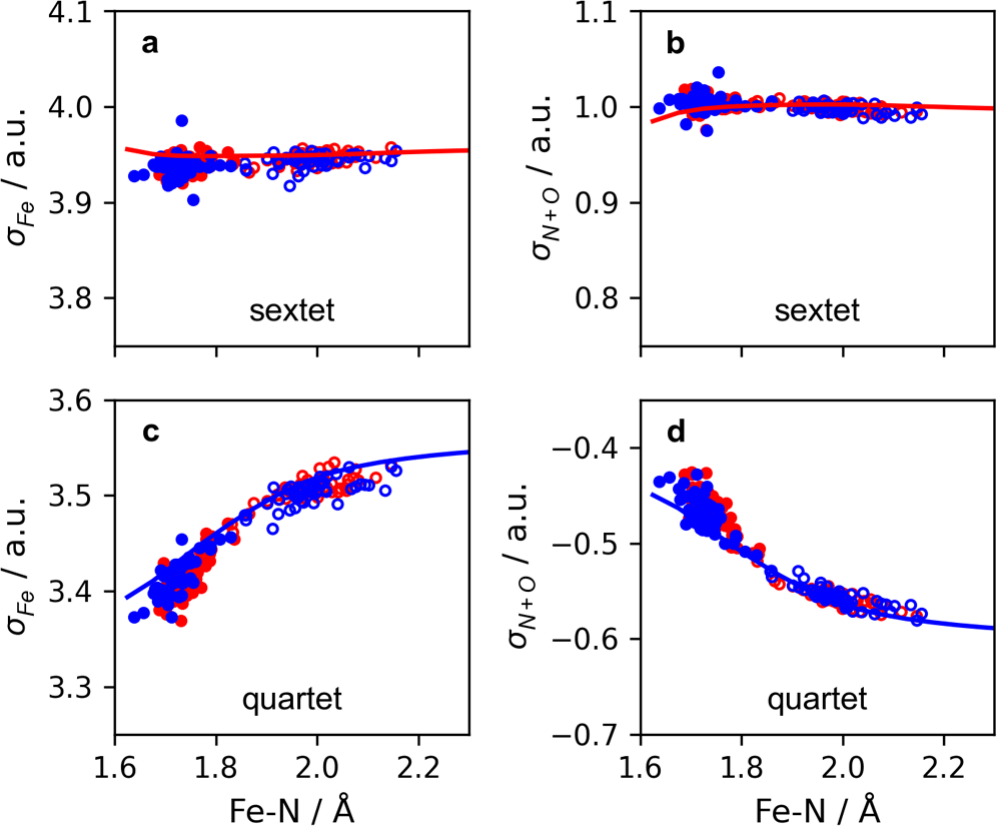
Using the coloring scheme described in Figure 8b, the iron σ_Fe_ atomic spin densities (a, c) and the combined nitrosyl σ_N+O_ atomic spin densities (b, d) are correlated with the Fe–N scan. The atomic spin densities obtained from the sextet calculations (top) and quartet calculations (bottom) are shown.

Lastly, we show the correlations of the sampled Mulliken atomic spin densities σ_Fe_ and σ_N+O_ with the changes of Fe–N distances in the liquid simulations by projecting all of the distributions in Figure 6a–f onto the model Fe–N coordinate in Figure 5d,f, respectively. In Figure 9, we show σ_Fe_ and σ_N+O_ for the sextet state calculated for each sampled geometry.

The distributions of the sampled properties in Figure 6d–f map well onto the model Fe–N reaction, showing that the sextet state is exclusively described by the  CSF, which places four unpaired electrons in the iron d-orbitals, giving σ_Fe_ of approximately 3.95 and one unpaired electron in the nitrosyl giving σ_N+O_ of approximately 1.0. In addition, the quartet σ_Fe_ and σ_N+O_ spin densities show clear trends that map to the Fe–N scan with some slight deviations at shorter Fe–N distances; however, we note that the general trends are preserved. While we display the Mulliken spin densities here, we provide the sampled Mulliken charges in Figure S16 and, for completeness, make a comparison to Voronoi deformation density (VDD) and Hirshfeld charge models in Figures S17 and S18, respectively.

### 3.4 Analysis Based on the CASSCF Wave Function

The interpretation of the multiconfigurational character of the quartet and sextet states was obtained from the CASSCF wave function by decomposing each state into the individual CSFs weighted by the squares of the coefficients determined variationally. The initial interpretation of the CSFs was taken from the quartet minimum at the optimized TPSSh structure. In ORCA, the identity and weight of these CSFs were tracked by a unique label initially assigned by the CASSCF routine, which is blind to the possible orbital rotations within the active space for each sampled geometry. Hence, deviations from the initial structure are subject to orbital rotations, which give the same CSF label but change the interpretation of the orbital composition in the active space. To understand the possible orbital rotations, Table S5 summarizes the shift in orbital ordering along the Fe–N scan at select geometries along the scan. At geometries between 1.72 and 2.32 Å in the scan, the first five orbitals in the active space show the rotation of the d_*xz*_ orbital at increasing Fe–N bond lengths. This shift has no effect on the identities of the leading CSFs in the CASSCF wave function and provides a basis for the sampled configurations to be interpreted in the same way. At large Fe–N distances in the scan (outside of the region containing the sampled structures), the wave function is dominated by a single CSF in both the quartet and sextet states. The orbital ordering of the initial  taken to be

(σ)^2^(π_*y*_)^2^(π_*x*_)^2^(d_*xz*_)^2^(d_*yz*_)^↑^(d_z^2^_)^↑^(d_*xy*_)^↑^(d_x^2^–*y*^2^_)^↑^(π_y_^*^)^↑^(π_x_^*^)^0^ involved the exchange of the π_y_^*^ and the d_*yz*_ orbitals and the overall shifting of the orbitals involved in the NO bonding to give (d_*xz*_)^2^(σ)^2^(π_*y*_)^2^(π_*x*_)^2^(π_y_^*^)^↑^(d_z^2^_)^↑^(d_*xy*_)^↑^(d_x^2^–*y*^2^_)^↑^(d_*yz*_)^↑^(π_x_^*^)^0^. This has no change on the interpretation of the wave function for the CSFs describing those states and could be written in any arbitrary order for the doubly occupied or for the singly occupied or for the unoccupied orbitals. We therefore consider this analysis along the Fe–N coordinate to be representative of the validity of the CSF weights obtained for the geometries in the configurational sampling (see Figure 8).

The Mulliken population analysis in Figures 5c–f and 9 provides insight into charges and spin densities of the multiconfigurational quartet state and how they differ from the sextet state described by a single CSF. CASSCF has been demonstrated by Radoń et al.^20^ and Boguslawski et al.^42^ to accurately describe {FeNO}^7^ spin densities in states described by single and multireference wave functions, with the latter study stressing the importance of the active space size. In the study by Rado et al., the authors considered a larger active space of CAS(9e,13o), which included an additional set of 4d orbitals and excluded the π_*x*_/π_*y*_ orbitals used in the current study. The authors used a BP86/def2-TZVP optimized “brown-ring” complex in *C*_s_ symmetry to give a linear (θ_Fe–N–O_ = 180°) moiety. They find that the quartet ground state wave function has a Mulliken atomic spin density of σ_Fe_ = 3.45 and σ_N+O_ = −0.49, which are nearly identical to the values given here. In our study, at large distances in the Fe–N scan, we see a plateaued value of σ_Fe_ = 3.55 rather than an expected value tending to be but not exactly equal to σ_Fe_ = 4.0. Freitag et al.^43^ identified a similar behavior of the Mulliken spin density with respect to a ruthenium nitrosyl complex, where they find the Mulliken population analysis to predict a spin density below the expected value, which the authors attribute to the mixing of electronic configurations in the wave function. The wave function at large Fe–N distances in the scan is described exclusively by , which contains four unpaired electrons residing on the iron. We therefore expected a similar behavior of the quartet state spin densities to that of the sextet spin densities at large Fe–N distances in the scan, both of which have an identical number of unpaired electrons on the iron and NO moieties. Similarly, the value of σ_N+O_ should tend to a value of roughly σ_N+O_ = −1.0 and instead has a plateaued value of σ_N+O_ = −0.55.

To understand this behavior, we turn our focus to the limitations of the Mulliken population analysis for the state-specific quartet CAS(13e,10o) wave function. In Table S6, we consider the [Fe(H_2_O)_5_]^2+^ and NO fragments taken from the TPSSh/def2-TZVP optimized “brown-ring” structure that was used to construct the rigid scan. Here, [Fe(H_2_O)_5_]^2+^ in a quintet state is represented by the wave function with a CAS(6e,5o) active space, while NO in a neutral doublet state is represented by the wave function with a CAS(7e,5o) active space. We show that the two active spaces preserve size consistency by having a difference in energy from the CAS(13e,10o) active space of ≈10^–5^ hartree, allowing us to make a comparison of the properties. The Mulliken population analysis for each fragment shows that the charges are correctly described in the “brown-ring” complex. Instead, the deviation is clear in the σ_Fe_ and σ_N+O_ spin densities for the quartet state. We find that the spin densities for the fragments have the correct values of σ_Fe_ ≈ 4.0 (actual σ_Fe_ = 3.95) and σ_N+O_ = 1.0 (actual σ_N+O_ = 1.0). This a clear explanation that the Mulliken population analysis has a limitation in describing the multiconfigurational wave function of the “brown-ring” complex at an increased Fe–N distance in the quartet state. We do not explore this phenomenon further but suggest that this limitation in the spin density analysis potentially extends to other multiconfigurational wave functions describing {FeNO}^7^ systems in their quartet states.

The identity of the oxidation state of iron as a quantitative measure is not central to this study, rather electronic fluctuations of the multiconfigurational wave function have been explored. Our analysis of the CASSCF wave function has been based on natural orbitals, which contain some level of orbital delocalization across the atoms in the {FeNO}^7^ moiety, namely, overlap between the d/π* orbitals. In order to quantify the ionic contributions from the CASSCF wave function, Radoń et al.^20^ localized the molecular orbitals in the active space. Using the localized orbitals, the authors constructed resonance structures based on individual atomic contributions, leading to a description of the “brown-ring” complex as predominantly Fe(II)–NO and Fe(III)–NO^–^. Based on the CSF weights in eq 2, the quartet ground state could be qualitatively described as being 53.9% Fe(I)–NO^+^, 26.1% Fe(II)–NO^0^, and 11.8% Fe(III)–NO^–^. Based on our results, we cannot comment on the exact description of the oxidation state, but we reflect that based on the multiconfigurational wave function, the qualitative description of the oxidation state is likely subject to change along the Fe–N scan.

## 4 Conclusions

The “brown-ring” complex is the simplest aqueous FeNO species and serves as an exemplar of many FeNO complexes. We feel that the results of this type of structural and electronic analysis are generally applicable to many important FeNO complexes, namely, those involved in biological systems (myoglobin, hemoglobin, etc.) and those possessing highly multiconfigurational electronic states. Through AIMD simulations and multiconfigurational quantum chemistry calculations, we have shown how the multiconfigurational description of the “brown-ring” complex is represented in bulk liquid. Due to the computational challenge of describing a multiconfigurational wave function by periodic DFT calculations, we have demonstrated that a structural sampling and recalculation of energies and properties at the CASSCF/NEVPT2 levels of theory give an elevated description of the electronic density around the FeNO moiety in the bulk liquid. Based on indications from the AIMD simulations, we identified that the multiplicity of the quartet and sextet states is correlated with the Fe–N distance. This provided the basis to construct a rigid Fe–N scan using CASSCF/NEVPT2, from which we extracted the NEVPT2 potential energies and the charges, spin densities, and weights of the electronic configurations based on the CASSCF wave function. We found that small changes in Fe–N distance produce large changes in the relative weights of the electronic configurations making up the quartet wave function. Interestingly, these changes accompany a large shift in spin density for the quartet state but correlate with a small change in charge. We find that the rise and decay of a doubly excited electronic configuration for the quartet state allow for neutral nitrosyl dissociation. By performing a structural sampling of the AIMD simulations, we find these trends of the electronic wave function to be preserved and hence are encoded within the bulk liquid.

## Data Availability

The data sets generated and analyzed during the current study are available from the corresponding author on reasonable request.

## Supporting Information Available

Tables of energies, table of optimized structure bond lengths and angles, Cartesian-optimized geometries, water-exchange benchmark energies, UKS potential energies, BLYP AIMD results, BP86 water-exchange mechanism, BLYP water-exchange mechanism, BP86 energies from the configuration sampling, BLYP energies from the configuration sampling, Fe–N–O bending angle scans, details of the CASSCF optimized relaxed scan, charge model analysis, CASSCF active space decomposition, and Mulliken analysis (PDF)
