# Supplementary material for: Simulations of the Aqueous “Brown-Ring” Complex Reveal Fluctuations in Electronic Character
Source: Inorg Chem. 2023 Oct 2;62(41):16854–66. doi: 10.1021/acs.inorgchem.3c02320 (PMC10583216; doi:10.1021/acs.inorgchem.3c02320)
Supplement: Supplementary file 1 — ic3c02320_si_001.pdf [file ic3c02320_si_001.pdf]

Supporting Information for:

Simulations of the aqueous "brown-ring" complex reveal fluctuations in electronic character.

Michael R. Coates ,[*,†] Ambar Banerjee ,[†,‡] and Michael Odelius [*,†]

†*Department of Physics, Stockholm University, AlbaNova University Center, SE-106 91 Stockholm Sweden*

‡*Department of Physics and Astronomy, Uppsala University, Box 516, SE-751 20 Uppsala, Sweden*

E-mail: michael.coates@fysik.su.se; odelius@fysik.su.se

S1 Quantum chemistry calculations

S1.1 Energies

Table S1: Absolute energies for the quartet, sextet and doublet computed for the TPSSh/def2-TZVP optimized quartet penta-aqua structure from Ref.[1] used as a reference in the current study.

State	quartet (kcal mol^{-1})	sextet (kcal mol^{-1})	doublet (kcal mol^{-1})
E (CASSCF)	-1772.012210	-1771.973950	-1771.933192
E (NEVPT2)	-1774.032802	-1773.985149	-1773.967746
E (TPSSh)	-1775.855569	-1775.812723	-1775.817782
E (BP86)	-1776.079951	-1776.022635	-1776.049083
E (BLYP)	-1775.835752	-1775.778037	-1775.808148

Table S2: Relative energies (kcal mol^{-1}) for the quartet, sextet and doublet states computed for the TPSSh/def2-TZVP optimized quartet penta-aqua structure from Ref.[1] used as a reference in the current study.

State	quartet (kcal mol^{-1})	sextet (kcal mol^{-1})	doublet (kcal mol^{-1})
E (CASSCF)	0.00	24.00	49.58
E (NEVPT2)	0.00	29.90	40.82
E (TPSSh)	0.00	26.89	23.71
E (BP86)	0.00	35.97	19.37
E (BLYP)	0.00	36.22	17.32

S1.2 Optimized structures

Table S3: The geometrical information of DFT optimized structures in ORCA version 5.0.1[2] using the TPSSh (meta-hybrid), BP86 (pure) and BLYP (pure) functionals for the $[Fe(H_2O)_5(NO)]^{2+}$ and $[Fe(H_2O)_4(NO)]^{2+}$ structures in the quartet and sextet states. Reproduction of the TPSSh structures optimized in the quartet state using Gaussian in Ref.[1] is shown in the top row, followed by the structures obtained in the present study.

Level of theory	$[Fe(H_2O)_5(NO)]^{2+}$			$[Fe(H_2O)_4(NO)]^{2+}$		
	Fe-NO angle (°)	Fe-N Å	N-O Å	Fe-NO angle (°)	Fe-N Å	N-O Å
TPSSh (Ref 1)	176.9	1.78	1.14	177.0	1.76	1.14
TPSSh (quartet)	179.3	1.77	1.14	179.9	1.76	1.14
TPSSh (sextet)	179.2	2.08	1.15	165.6	2.05	1.15
BP86 (quartet)	165.4	1.75	1.15	179.95	1.73	1.15
BP86 (sextet)	179.1	2.01	1.17	166.8	1.98	1.16
BLYP (quartet)	166.4	1.77	1.15	179.9	1.75	1.15
BLYP (sextet)	179.2	2.05	1.17	166.6	2.02	1.17

TPSSh quartet penta-aqua minimum (units Å)

TPSSh/def2-TZVP CPCM(water) optimized

E(hartree) = -1775.857698982164

Fe	-0.03826	0.0194	-0.093788
O	-0.08366	2.101958	-0.302672
O	2.079391	0.005248	-0.46766
O	-0.05582	0.142889	-2.24628
O	-0.07616	-2.059746	-0.352389
O	-2.153788	0.009671	-0.436675
H	2.549053	0.829187	-0.261382
H	2.579608	-0.699969	-0.027146
H	0.143393	2.471049	-1.170308
H	0.258724	2.709341	0.370349
H	-0.841129	-0.166426	-2.722169
H	0.717208	-0.171407	-2.738778
H	-2.647812	-0.701987	-0.000144
H	-2.614877	0.832819	-0.209481
H	0.172413	-2.446483	-1.206122
H	0.251262	-2.660005	0.334925
N	-0.024125	-0.006187	1.678652
O	-0.015421	-0.009352	2.821167

TPSSh sextet penta-aqua minimum (units Å)

TPSSh/def2-TZVP CPCM(water) optimized

E(hartree) = -1775.830550610448

Fe	-0.026703	0.03126	-0.271826
O	-0.0771	2.120859	-0.365514
O	2.058137	0.035025	-0.353487
O	-0.017434	0.159669	-2.421916
O	-0.062716	-2.061693	-0.388604
O	-2.106876	0.020898	-0.333168
H	2.54565	0.83086	-0.088276
H	2.552784	-0.725403	-0.009525
H	0.14273	2.550316	-1.20618
H	0.213771	2.712133	0.344428
H	-0.849939	-0.041247	-2.876643
H	0.672637	-0.328123	-2.896884
H	-2.588462	-0.767624	-0.037726
H	-2.596988	0.792222	-0.007858
H	0.145405	-2.518984	-1.217405
H	0.213643	-2.645078	0.333788
N	-0.012022	0.014254	1.807719
O	-0.006519	0.020656	2.959177

TPSSh quartet tetra-aqua minimum (units Å)

TPSSh/def2-TZVP CPCM(water) optimized

E(hartree) = -1699.371745572963

Fe	-0.027591	0.026051	0.215359
O	-0.081629	2.060549	-0.149706
O	1.859633	0.029361	-0.62351
O	-0.079594	-2.006392	-0.159087
O	-1.909529	0.026847	-0.617692
H	2.423521	0.808668	-0.491439
H	2.425553	-0.748906	-0.494094
H	0.089888	2.423642	-1.033328
H	0.253358	2.704363	0.493824
H	-2.471627	-0.754502	-0.491402
H	-2.473715	0.806134	-0.488078
H	0.092743	-2.366191	-1.043905
H	0.255233	-2.652479	0.482266
N	0.002363	0.021135	1.970695
O	0.021133	0.016665	3.107425

TPSSh sextet tetra-aqua minimum (units Å)

TPSSh/def2-TZVP CPCM(water) optimized

E(hartree) = -1699.340527137631

Fe	-0.024406	0.025958	0.092307
O	-0.065553	2.093397	-0.018416
O	1.807818	0.0298	-0.765738
O	-0.062761	-2.04066	-0.028161
O	-1.868279	0.027033	-0.751431
H	2.378838	0.814476	-0.757025
H	2.380408	-0.753774	-0.761068
H	0.07586	2.56872	-0.852142
H	0.232913	2.675774	0.696577
H	-2.439804	-0.757012	-0.734012
H	-2.440256	0.810667	-0.730834
H	0.078841	-2.511508	-0.864399
H	0.237378	-2.626134	0.683609
N	0.155047	0.021083	2.139218
O	-0.032299	0.01815	3.270543

BP86 quartet penta-aqua minimum (units Å)

BP86/def2-TZVP CPCM(water) optimized

E(hartree) = -1776.083650343609

Fe	-0.081753	0.015562	-0.084545
O	-0.122108	2.100784	-0.312143
O	2.063121	-0.006	-0.403838
O	-0.038339	0.153372	-2.256842
O	-0.104589	-2.074333	-0.367918
O	-2.214932	0.001305	-0.502765
H	2.510962	0.838307	-0.195683
H	2.534885	-0.682469	0.121495
H	0.157386	2.431752	-1.188352
H	0.249411	2.706242	0.35794
H	-0.813256	-0.196105	-2.736655
H	0.751112	-0.190161	-2.716853
H	-2.706248	-0.694407	-0.023544
H	-2.663022	0.839189	-0.272665
H	0.237739	-2.435288	-1.208659
H	0.222893	-2.663064	0.33946
N	-0.043262	-0.007665	1.662085
O	0.262247	0.049216	2.76979

BP86 sextet penta-aqua minimum (units Å)

BP86/def2-TZVP CPCM(water) optimized

E(hartree) = -1776.038975742624

Fe	-0.033827	0.034305	-0.23751
O	-0.085453	2.123566	-0.36423
O	2.061257	0.026328	-0.373376
O	-0.031163	0.174902	-2.400815
O	-0.075032	-2.058101	-0.399012
O	-2.123382	0.021429	-0.346851
H	2.546788	0.827466	-0.093099
H	2.546897	-0.736474	-0.001572
H	0.153988	2.536329	-1.216469
H	0.237457	2.713375	0.343052
H	-0.853093	-0.104185	-2.84894
H	0.694611	-0.286843	-2.863425
H	-2.599055	-0.769478	-0.024766
H	-2.609755	0.799571	-0.009896
H	0.167521	-2.500392	-1.235224
H	0.223253	-2.640416	0.325395
N	-0.014171	0.016013	1.774165
O	-0.006842	0.022609	2.942674

BP86 quartet tetra-aqua minimum (units Å)

BP86/def2-TZVP CPCM(water) optimized

E(hartree) = -1699.591813109479

Fe	-0.034154	0.025974	0.223346
O	-0.092742	2.067729	-0.15355
O	1.859356	0.02961	-0.640242
O	-0.090102	-2.014085	-0.162795
O	-1.921215	0.026727	-0.632531
H	2.422005	0.812019	-0.471807
H	2.423788	-0.752254	-0.475355
H	0.109348	2.420789	-1.042651
H	0.265435	2.705879	0.493869
H	-2.482988	-0.757941	-0.47336
H	-2.483736	0.810216	-0.470192
H	0.111837	-2.363425	-1.053391
H	0.268074	-2.654948	0.481938
N	0.000628	0.021284	1.954569
O	0.024207	0.017372	3.099482

BP86 sextet tetra-aqua minimum (units Å)

BP86/def2-TZVP CPCM(water) optimized

E(hartree) = -1699.542475299756

Fe	-0.033494	0.025947	0.159529
O	-0.083516	2.093509	0.005497
O	1.759635	0.029833	-0.778706
O	-0.08065	-2.040724	-0.004155
O	-1.82633	0.027152	-0.796087
H	2.335954	0.819987	-0.763915
H	2.337715	-0.759101	-0.767482
H	0.063391	2.549032	-0.846809
H	0.250993	2.683933	0.707617
H	-2.404032	-0.76171	-0.776034
H	-2.404487	0.815593	-0.773007
H	0.066137	-2.491985	-0.858738
H	0.255259	-2.63403	0.694871
N	0.164223	0.021004	2.1315
O	0.012947	0.017529	3.284947

BLYP quartet penta-aqua minimum (units Å)

BLYP/def2-TZVP CPCM(water) optimized

E(hartree) = -1775.839812126123

Fe	-0.010667	-0.019007	-0.093768
O	0.01484	2.100452	-0.247339
O	2.166592	0.043433	-0.255452
O	0.193692	-0.036928	-2.285385
O	0.08219	-2.113616	-0.43443
O	-2.140664	-0.005551	-0.659516
H	2.597099	0.689356	0.340811
H	2.598758	-0.814807	-0.067583
H	-0.204077	2.519128	-1.103711
H	-0.382695	2.659669	0.449367
H	-0.559837	0.304696	-2.805495
H	0.999518	0.363919	-2.665859
H	-2.625697	-0.819721	-0.414613
H	-2.652542	0.728425	-0.263269
H	-0.122792	-2.418883	-1.340782
H	-0.311832	-2.758652	0.185539
N	-0.100269	-0.07016	1.670628
O	0.091236	0.003948	2.805443

BLYP sextet penta-aqua minimum (units Å)

BLYP/def2-TZVP CPCM(water) optimized

E(hartree) = -1775.797355512228

Fe	-0.033052	0.033561	-0.239099
O	-0.083817	2.145509	-0.353803
O	2.077601	0.027819	-0.377932
O	-0.030735	0.171811	-2.429819
O	-0.073107	-2.080899	-0.38667
O	-2.138904	0.021545	-0.350053
H	2.568883	0.826671	-0.097353
H	2.569898	-0.741974	-0.026971
H	0.148432	2.570798	-1.20303
H	0.231914	2.733047	0.360186
H	-0.852508	-0.108446	-2.879794
H	0.698101	-0.286932	-2.892898
H	-2.622504	-0.769863	-0.03786
H	-2.63177	0.802414	-0.0259
H	0.161298	-2.528366	-1.223789
H	0.224933	-2.662569	0.339938
N	-0.011591	0.018808	1.811965
O	-0.003074	0.027065	2.982982

BLYP quartet tetra-aqua minimum (units Å)

BLYP/def2-TZVP CPCM(water) optimized

E(hartree) = -1699.368151075148

Fe	-0.035455	0.02588	0.23287
O	-0.090641	2.090394	-0.144234
O	1.870745	0.02975	-0.652528
O	-0.087964	-2.036855	-0.153476
O	-1.935895	0.026782	-0.643822
H	2.436615	0.813823	-0.496697
H	2.438601	-0.75357	-0.500242
H	0.109993	2.444532	-1.034581
H	0.267696	2.729531	0.503993
H	-2.501997	-0.759011	-0.499535
H	-2.502462	0.81169	-0.496527
H	0.112637	-2.387364	-1.045256
H	0.27018	-2.678681	0.492194
N	0.001375	0.021022	1.983638
O	0.026311	0.017021	3.131533

BLYP sextet tetra-aqua minimum (units Å)

BLYP/def2-TZVP CPCM(water) optimized

E(hartree) = -1699.321255704059

Fe	-0.027437	0.026148	0.150379
O	-0.073064	2.117549	0.007052
O	1.779836	0.029749	-0.792377
O	-0.07023	-2.064506	-0.002725
O	-1.846665	0.027047	-0.784307
H	2.355776	0.821464	-0.802778
H	2.357119	-0.760939	-0.806228
H	0.081808	2.582334	-0.840049
H	0.245206	2.70563	0.72024
H	-2.423645	-0.763835	-0.781928
H	-2.424508	0.817281	-0.778801
H	0.084489	-2.524839	-0.852282
H	0.249809	-2.655624	0.707161
N	0.147592	0.021123	2.161112
O	-0.022341	0.017388	3.314558

S1.3 Water exchange with different functionals

Table S4: Absolute energies, in hartrees, of the quartet energies of $[Fe(H_2O)_5(NO)]^{2+}$ and $[Fe(H_2O)_4(NO)]^{2+}$ + H_2O and the difference in energy between the two species. For $[Fe(H_2O)_4(NO)]^{2+}$ a DFT optimized H_2O energy (one for each functional) is added to make the energies comparable.

Species	$[Fe(H_2O)_5(NO)]^{2+}$ (hartree)	$[Fe(H_2O)_4(NO)]^{2+}$ + H_2O (hartree)	Δ E (hartree)
E (TPSSh)	-1775.85770	-1775.83814	0.01956
E (BP86)	-1776.08365	-1776.06694	0.01672
E (BLYP)	-1775.83981	-1775.82406	0.01575

S2 AIMD simulation of $[Fe(H_2O)_5(NO)]^{2+}$ in water

S2.1 UKS potential energies

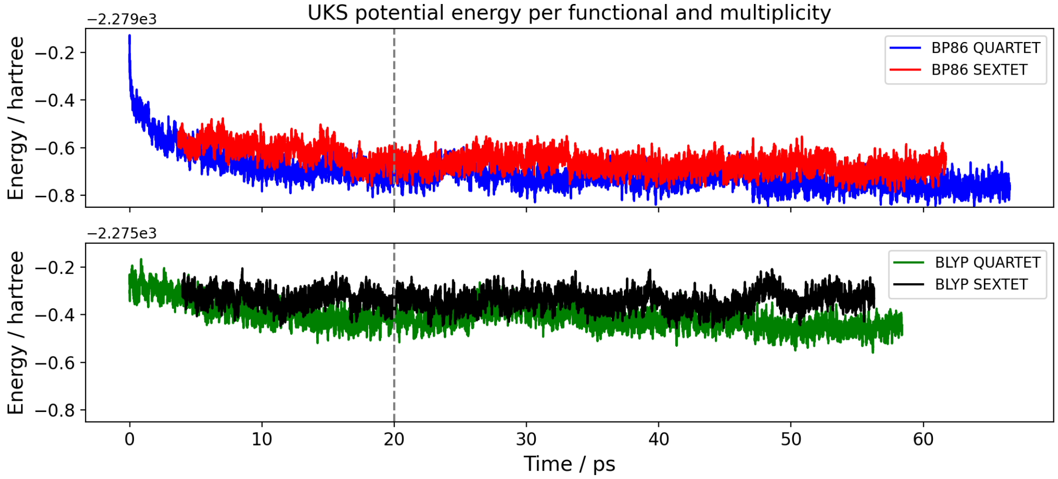

Figure S1: The UKS potential energies of the BLYP and BP86 AIMD trajectories for the total simulation. The vertical line is displayed to indicate the point at which the production run of each simulation was initiated.

S2.2 BLYP AIMD results

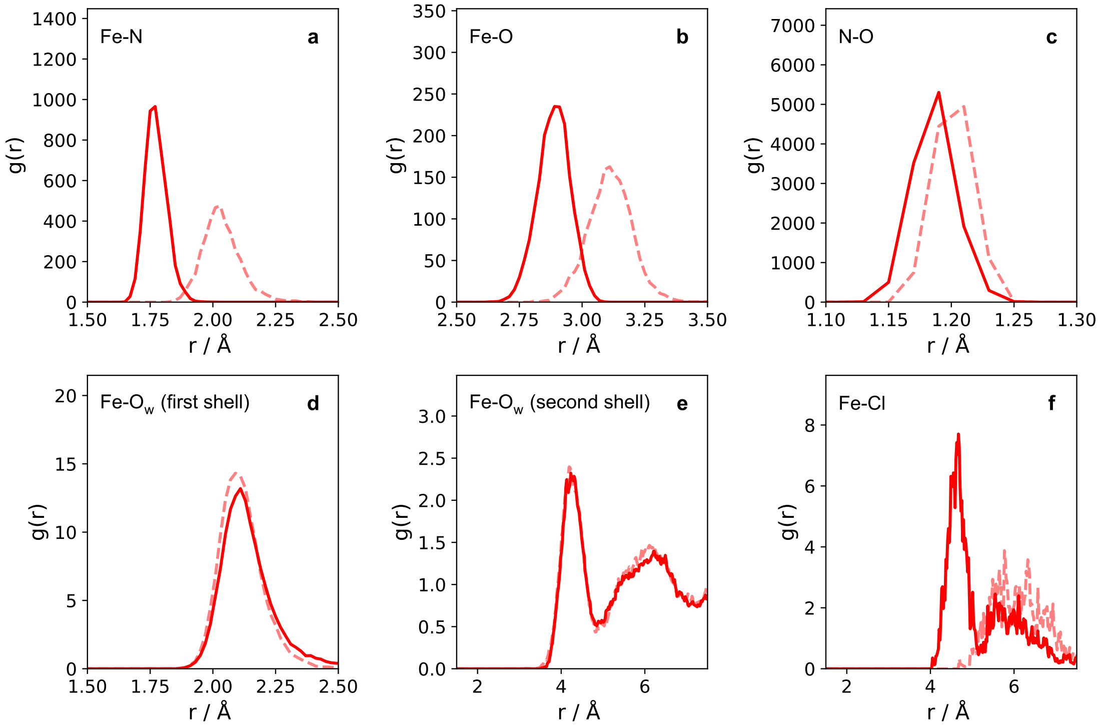

Figure S2: Radial distribution functions g(r) sampled from the BLYP AIMD trajectories of quartet and sextet simulations for distances between different pairs of atoms. (a) Fe-N (b) Fe-O_N (c) N-O (d) first shell Fe-O_w (e) second shell Fe-O_w (f) Fe-Cl. The quartet simulations are represented as solid lines, while the sextet simulations are represented by the opaque dashed lines. The red color for the penta-aqua coordination is used for both multiplicities, noting the non-existence of tetra-aqua coordination in the BLYP simulations.

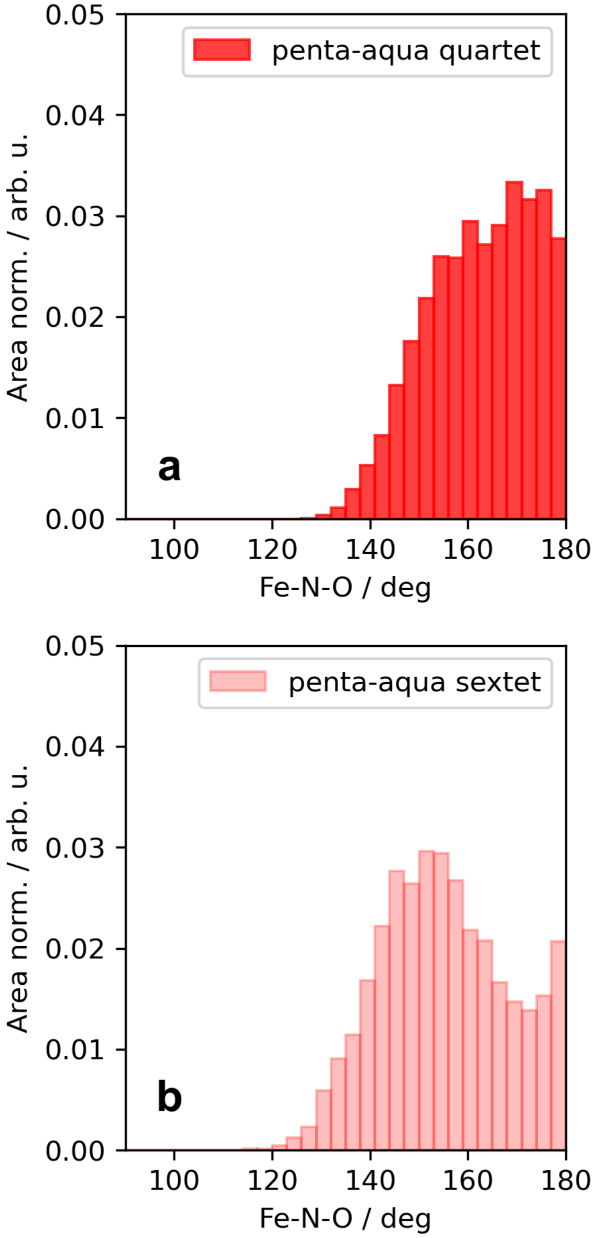

Figure S3: The distribution of angles from the BLYP AIMD trajectories. The angles sampled from the penta-aqua are colored in red with the quartet trajectory in (a) and the sextet trajectory in (b). All angle distributions are area normalized to compare peak intensities.

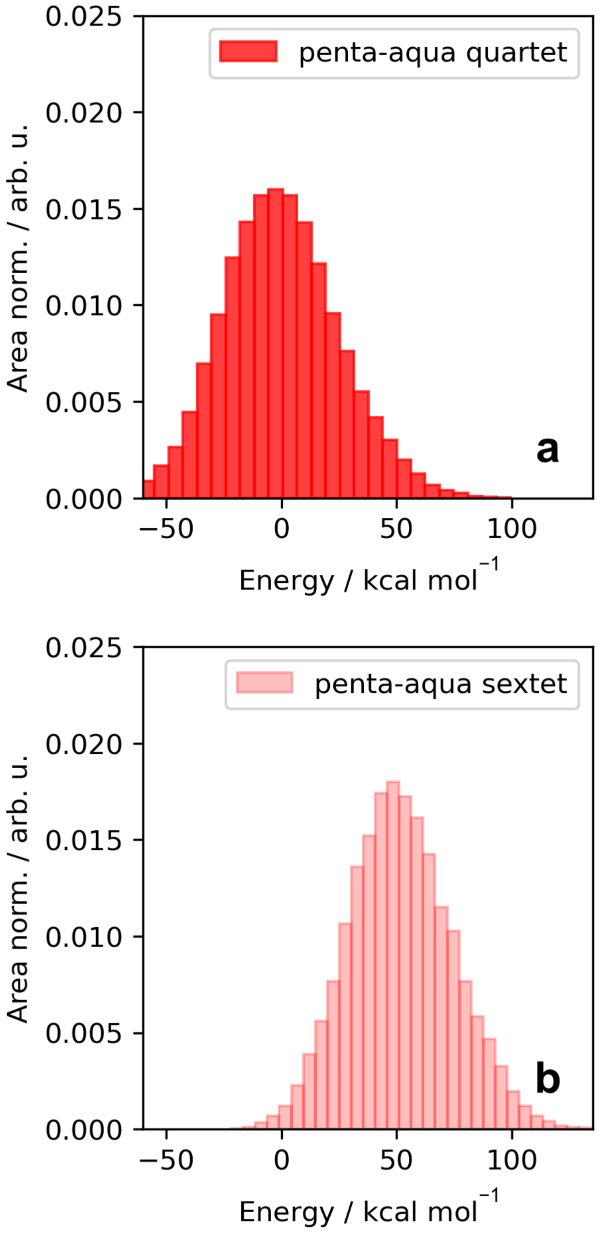

Figure S4: The distribution of potential (UKS) energies from the (a) quartet and (b) sextet BLYP AIMD trajectories. The energies in the penta-aqua trajectory. All energy distributions are area normalized to compare peak intensities. The energy distributions are referenced relative to the average potential in the full quartet trajectory.

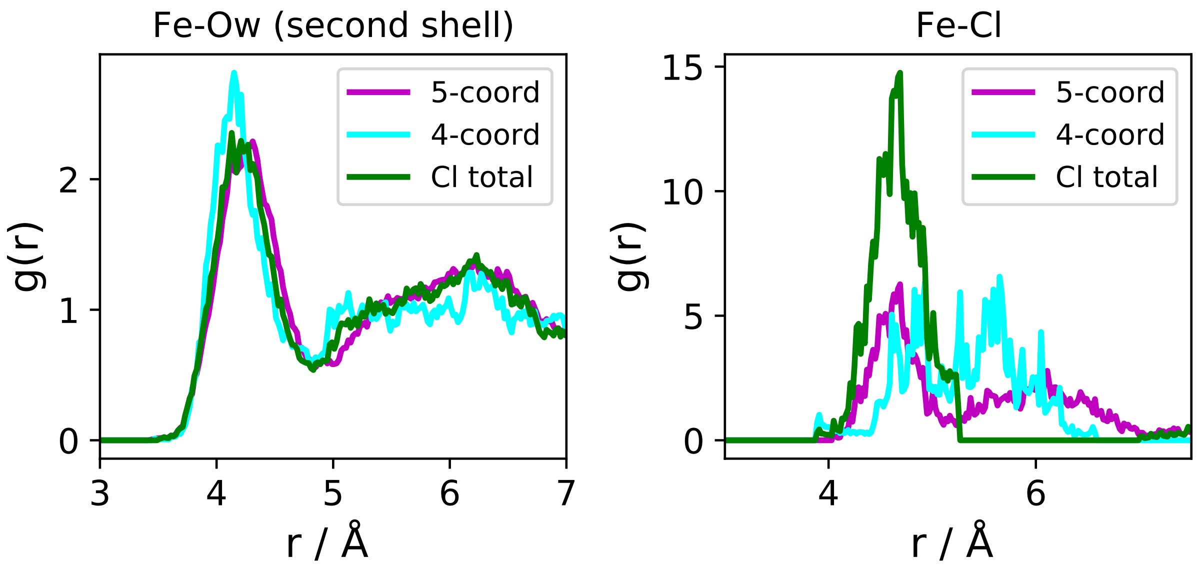

Figure S5: Radial distribution function g(r) for Fe-Cl sampled from the BLYP AIMD quartet trajectory with a comparison made between the penta-aqua and tetra-aqua sets of configurations.

S2.3 Water exchange with BP86

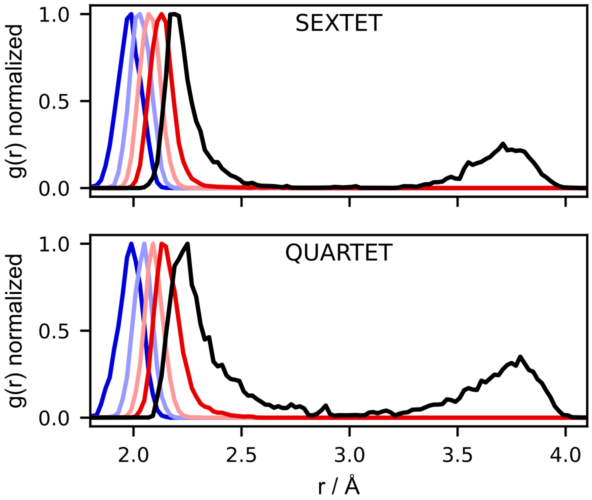

Figure S6: Comparison of the fifth water in the quartet and sextet BP86 AIMD simulation. Here the individual RDFs are normalized to highlight the exchange of the fifth coordinating water with the second solvation shell. Note that the quartet RDFs in the bottom panel are reproduced from Ref.[1].

S2.4 Water exchange in BLYP

We noted in the main text that there exists no indications of a dynamical equilibrium being established between the penta-aqua and tetra-aqua species during the production run using the BLYP functional. We found that during the initial 10-20 ps of the equilibration in the BLYP quartet simulation, there was an exchange of a water ligand resulting in the formation of the tetra-aqua species. In Fig. S7, we show the RDFs for the closest five water ligands in the quartet and sextet BLYP AIMD simulations and find that there is a slight bump in the fifth water RDF from the quartet state, centered around 3.8 Å, while no such peak exists in the sextet state.

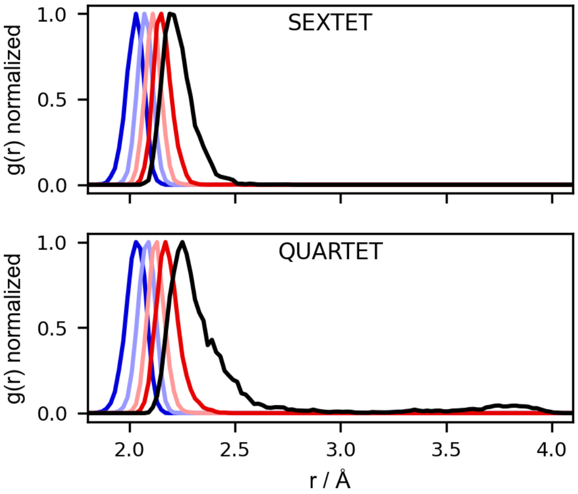

Figure S7: Comparison of the fifth water in the quartet and sextet BLYP AIMD simulation. Here the individual RDFs are normalized to highlight the exchange of the fifth coordinating water with the second solvation shell that occurs in the quartet state.

S2.5 BP86 configuration sampling

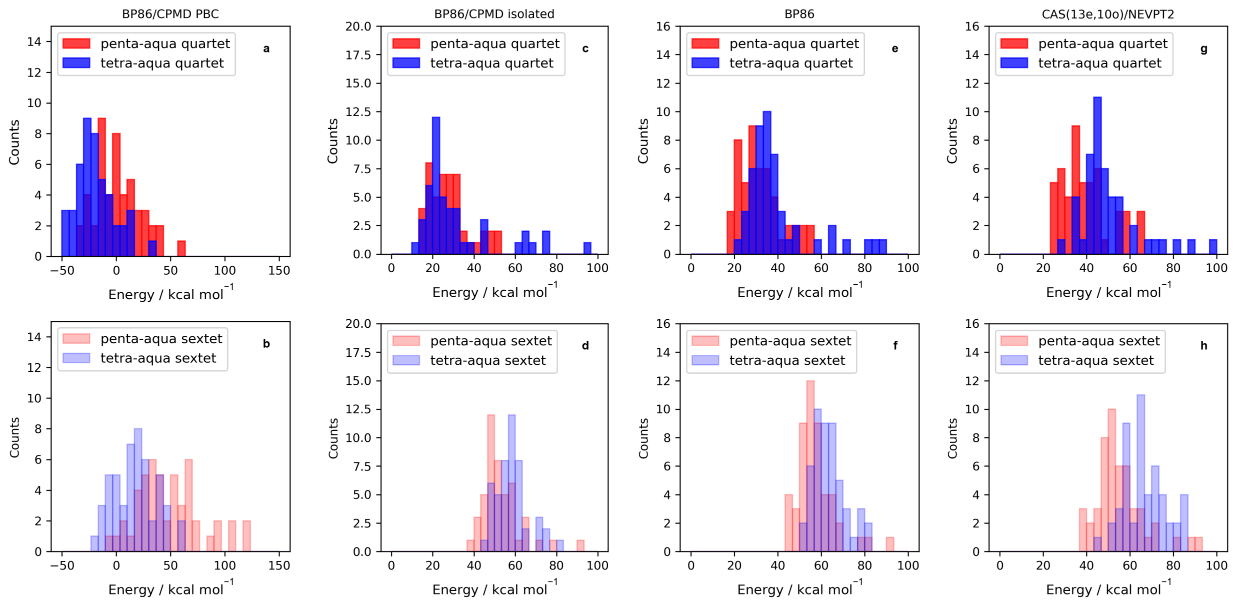

Figure S8: (a/b) The distributions of the CPMD BP86 energies corresponding to the bulk liquid simulation taken for each sampled complex from the four sets of configurations. The energies are shifted with respect to the average potential energy of the penta-aqua quartet distribution. (c/d) The distributions of the corresponding isolated CPMD BP86 energies for the sampled complexes from the four sets of configurations. The energies are shifted with respect to the CPMD BP86 isolated energy at the TPSSh quartet optimized structure. (e/f) The distributions of the BP86/def2-TZVP CPCM(water) energies for the sampled complexes from the four sets of configurations. The energies are shifted with respect to the BP86/def2-TZVP CPCM(water) energy at the TPSSh quartet optimized structure. (g/h) The distributions of the SA2-CAS(13e,10o)/NEVPT2 CPCM(water) energies for the sampled complexes from the four sets of configurations. The energies are shifted with respect to the SA2-CAS(13e,10o)/NEVPT2 CPCM(water) energy at the TPSSh quartet optimized structure.

S2.6 BLYP configuration sampling

To evaluate this exchange process, we repeat the analysis performed on the quartet and sextet BP86 simulations in Fig. S8 for the quartet BLYP simulation. We extracted 15 penta-aqua and 15 tetra-aqua configurations from the quartet BP86 simulation and display the corresponding bulk liquid CPMD BLYP energies in Fig. S9.

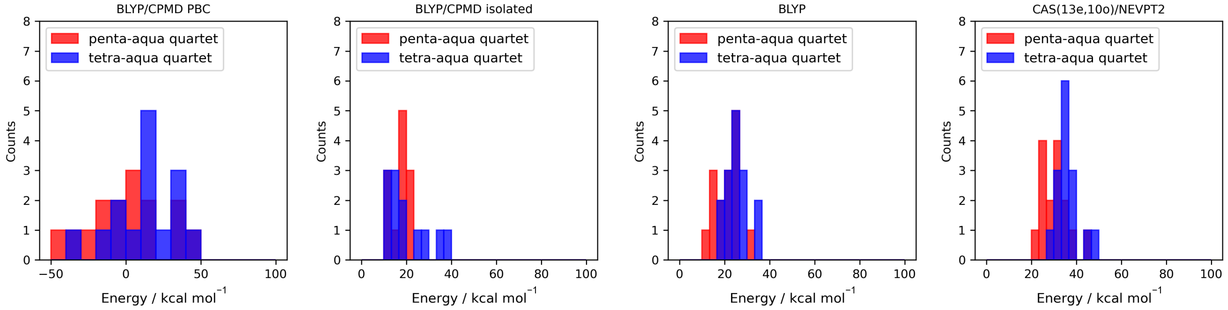

Figure S9: (a) The distributions of the CPMD BLYP energies corresponding to the bulk liquid simulation taken for each sampled complex from the two sets of quartet configurations. The energies are shifted with respect to the average potential energy of the penta-aqua quartet distribution. (b) The distributions of the corresponding isolated CPMD BLYP energies for the sampled complexes from the two sets of quartet configurations. The energies are shifted with respect to the CPMD BLYP isolated energy at the TPSSh quartet optimized structure. (c) The distributions of the BLYP/def2-TZVP CPCM(water) energies for the sampled complexes from the two sets of quartet configurations. The energies are shifted with respect to the BLYP/def2-TZVP CPCM(water) energy at the TPSSh quartet optimized structure. (d) The distributions of the SA2-CAS(13e,10o)/NEVPT2 CPCM(water) energies for the sampled complexes from the two sets of quartet configurations. The energies are shifted with respect to the SA2-CAS(13e,10o)/NEVPT2 CPCM(water) energy at the TPSSh quartet optimized structure.

We find that the tetra-aqua species in BLYP to be on average higher in energy than the penta-aqua sampled energies, however, we note that these were taken from the equilibration part of the trajectory and hence the energies are not as reliable. We instead find a nice agreement along all isolated cluster energies calculated by BLYP/CPMD, BLYP and NEVPT2, which show that the energies between the penta-aqua and tetra-aqua species are overlapping, indicating an enthalpic argument for the possibility of this species occurring in the liquid simulations. We note that due to the short AIMD simulations, we cannot rule out

the possibility of such an equilibrium being established in a longer simulation.

S2.7 Validity of the AIMD simulations

We highlight the possible bias of the structures used in the configurational sampling that occurs as a result of the choice of DFT functional. The use of DFT to describe $\{FeNO\}^7$ complexes has been detailed,[3–5] where the relative bond lengths and angles have shown a systematic bias with the choice of functional type (pure, hybrid, range-separated etc.). The FeNO bending angle described in Fig. 3 showed the structural dependence with the multiplicity and the solvation of the FeNO moiety. Here the penta-aqua and tetra-aqua distributions showed a reverse ordering between the quartet and sextet sets of configurations. In Fig. S10(a/b) and Fig. S10(c/d) we display rigid scans of the bending angles in each of the four types of configurations displayed in Fig. 3.

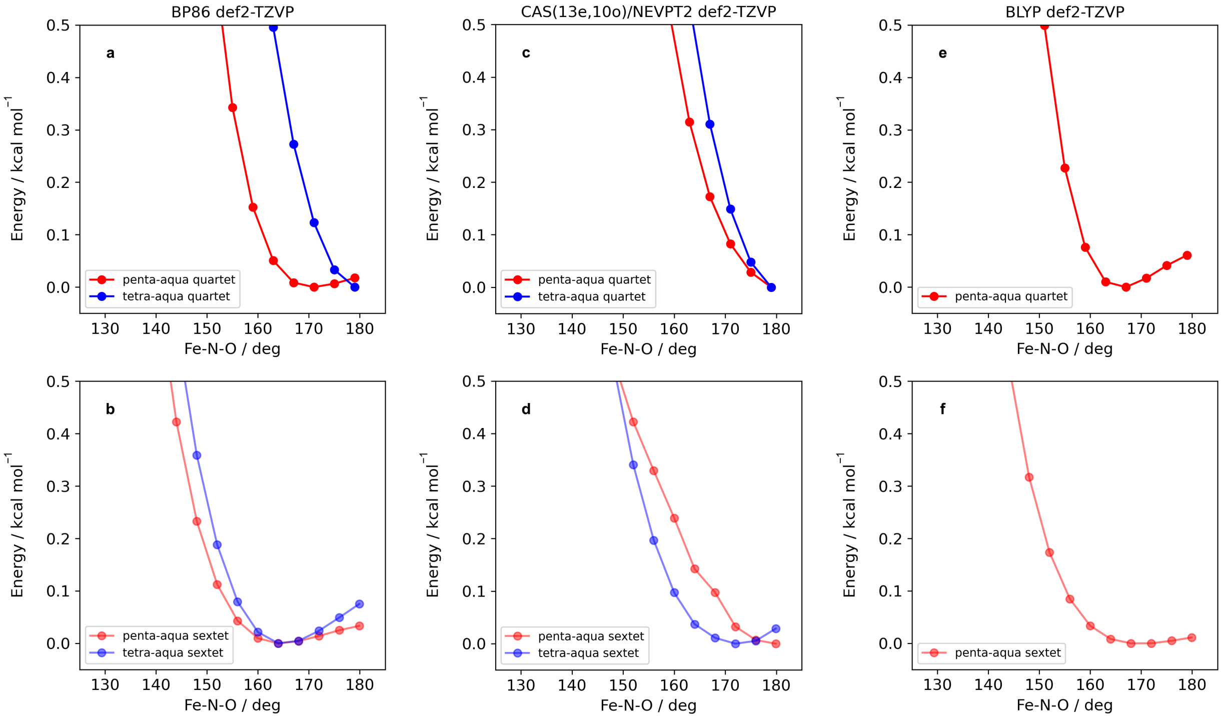

Figure S10: Rigid scans along the Fe-N-O bending angle calculated using BP86/def2-TZVP (a/b), CAS(13e,10o)/NEVPT2 def2-TZVP (c/d) and BLYP def2-TZVP (e/f). (a/c/e) correspond to the quartet states and (b/d/f) correspond to the sextet states. The starting structures for each rigid scan were taken from the TPSSh/def2-TZVP optimized structures.

The rigid scans starting from structures optimized at the TPSSh/def2-TZVP level of theory were re-calculated at the BP86/def2-TZVP (Fig. S10(a/b)) and at the SA(1Q+1S)-

CAS(13e,10o)/NEVPT2 (Fig. S10(c/d)) levels of theory. The rigid scan in Fig. S10(a) shows preferentially linear FeNO angle in the tetra-aqua quartet surface when compared to the penta-aqua quartet surface which adopts a slightly bent structure at 173°. This difference appears to be reflected in the ordering of the distributions in Fig. 3(a). The rigid scans in Fig. S10(b) prove to be less insightful, with the difference between penta-aqua sextet surface and tetra-aqua sextet surface being less distinct, as they are overlapping in majority of the scan. The distributions of the FeNO angle from the AIMD simulations using the BLYP functional in Fig. S3 indicate that there is a preference for a linear FeNO angle in the penta-aqua quartet complex (Fig. S3(a)) when compared to the penta-aqua sextet complex (Fig. S3(b)). Rigid scans starting from structures optimized at the TPSSh/def2-TZVP level of theory and re-calculated at the BLYP/def2-TZVP in Fig. S10(e/f) indicate that based on the potential energy surfaces alone, the penta-aqua sextet complex would instead be more linear. We note, however, that the minima of these surfaces is shallow (0.05 kcal mol^{-1}) like the surfaces calculated for the BP86 functional in Fig. S10(a/b) and at the NEVPT2 level of theory in Fig. S10(c/d). We find that between both functionals, the differences in the energies (0.5 kcal mol^{-1}) are much less than the chemical accuracy of the DFT calculations. We stress that these fluctuations in bending angle are likely related to the choice of functional as evidenced by the literature. However, we can only refer to the literature for the penta-aqua quartet case, as the existence of the tetra-aqua species is recent[1] and the sextet structures have not been explored in any significant detail.[3–5]

The dynamics simulated in the sextet multiplicity have a possible limitation related to the presence of quasi-degenerate sextet states. In Fig. 5(a), these states are shown in opaque red in the sextet minimum at an Fe-N distance of 2.1 Å. The AIMD simulations were simulated according to the Born-Oppenheimer approximation which neglected the couplings to other adiabatic electronic states, thus confining the dynamics to the lowest energy sextet state accessed at the start of the simulation. We neglect these additional states for the sake of simplicity since they appear to be parallel to the lowest energy sextet state along the Fe-N

scan in Fig. 5(a). As a result, these additional states would likely yield similar sampled configurations, particularly those along the desired Fe-N scan. We note that the ability for the simulation to sample all sextet states would properly sample more configuration space and as a consequence the configurations inherently have a possible bias from our approximation. The clear energetic separation of the UKS potential energy distributions in Fig. 4 prompts a short discussion on the nature of the water exchange equilibrium. The AIMD energies in both multiplicities suggest the energetic favourability of the the dynamical equilibrium between the penta-aqua and tetra-aqua species. We note in Ref. 1, that BP86 predicts a square pyramidal tetra-aqua complex roughly 4 kcal mol^{-1} lower than the hybrid TPSSh functional and 5 kcal mol^{-1} lower in energy than a NEVPT2/CASPT2 calculation. We previously stressed the validity of such an equilibrium, where here we stress the importance of the BP86 functional in the formation of the relative shifts of the UKS potential energy distributions in Fig. 4. In Fig. S8(a/b) we display the distributions of the CPMD BP86 UKS potential energies that correspond to each sampled point from the four sets of configurations. These distributions therefore contain a subset of energies in Fig. 4 and we find that the ordering of the distributions is the same, ruling out a possible bias from the sampling. In Fig. S8(c-h) we find that the ordering of potential energy distributions is reversed when the isolated clusters are calculated at different levels of theory. In Fig. S8(c/d), distributions of the CPMD BP86 UKS potential energies of the isolated clusters show a preference for the penta-aqua species when compared to the corresponding configurations of the bulk liquid. Based on this, we note that the effect of the pseudopotentials and plane wave basis have little influence on the existence of the tetra-aqua species, instead, long range interactions of the liquid are responsible for the observed equilibrium. The distributions of the BP86/def2-TZVP CPCM(water) calculations in Fig. S8(e/f) indicate an energetic overlap between the penta-aqua and tetra-aqua species, with the penta-aqua species being preferred. This is in agreement with the implicitly solvated structures in our previous study,[1] which indicated a small energetic difference between penta-aqua and tetra-aqua species and

an overall preference for the penta-aqua species. Lastly, we show the distributions of the SA(1Q+1S)-CAS(13e,10o)/NEVPT2 CPCM(water) potential energies in Fig. S8(g/h) which are in agreement with the isolated DFT distributions in Fig. S8(c-f). Based on this, we conclude that the bulk liquid interactions show an energetic preference for the tetra-aqua species in both the quartet and sextet multiplicities, while the isolated clusters reflect the opposite trend.

We have detailed the role of the multiconfigurational wavefunction in the description of the quartet and sextet ground states. We note that the AIMD simulations based on the single-reference DFT wavefunction are therefore a limitation in the analysis of the “brown-ring” complex. In order to capture some of the multiconfigurational character, the underlying electronic structure of the simulations were performed in with an unrestricted Kohn-Sham DFT approach. We previously discussed the the approximation of the effects due to multi-configurational wavefunction and validity of the UKS simulations in Ref. 1. To expand on the previous analysis, we consider the BP86 energies for the relaxed and rigid Fe-N scans in Fig. S11. The grey region in Fig. S11(a/b) indicates the maximum and minimum Fe-N distances sampled by the quartet AIMD simulation. We find that the BP86 energies are in close agreement with NEVPT2 results within the vicinity of the quartet minimum, indicating that the Fe-N distances are properly described by BP86 in comparison with a multiconfigurational wavefunction. We find the use of BP86 as an approximation to the multiconfigurational wavefunction to be appropriate in the context of this study on the basis that we use the simulations to generate structures for our configurational sampling. We subsequently re-calculated the electronic properties of the sampled configurations at a more appropriate level of theory.

S3 Fe-N model reaction

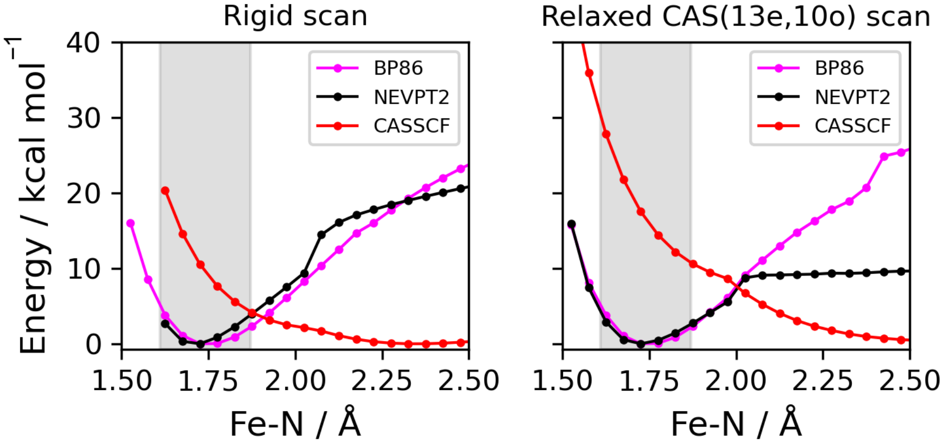

Figure S11: The BP86 and NEVPT2 energies along the rigid scan taken from the penta-aqua TPSSh/def2-TZVP optimized structure and relaxed scan along Fe-N distances (1.52 Å to 3.02 Å) optimized at the SS(1Q)-CAS(13e,10o)/def2-TZVP+cpcm(water) level of theory. (a) Shows the NEVPT2 and BP86 energies for the rigid scan. (b) Shows the NEVPT2 and BP86 energies for the relaxed scan. The grey shaded region denotes the maximum and minimum Fe-N distances sampled by the quartet AIMD simulation.

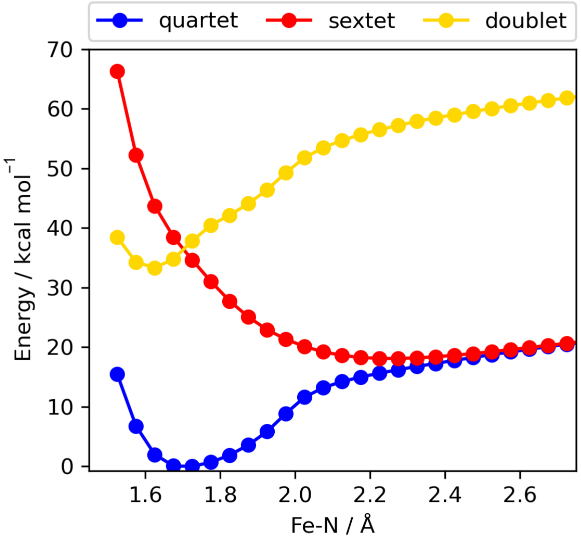

Figure S12: The potential energy curves of the quartet (blue), sextet (red) and doublet (yellow) ground states for the rigid scan taken from the penta-aqua TPSSh/def2-TZVP optimized structure.

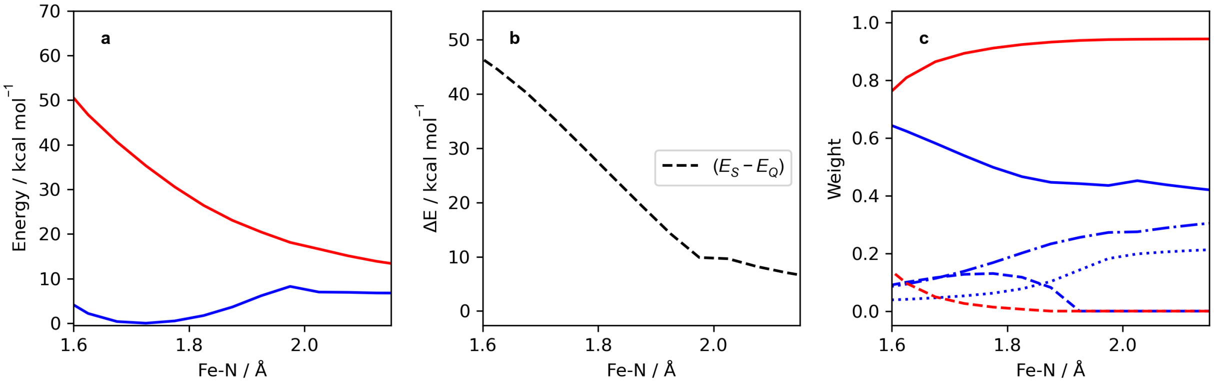

Figure S13: Relaxed scan along Fe-N distances (1.52 Å to 3.02 Å) optimized at the SS(1Q)-CAS(13e,10o)/def2-TZVP+cpcm(water) level of theory. (a) Shows the corresponding SA2(1Q+1S)-CAS(13e,10o)/NEVPT2 energies along the relaxed scan. (b) shows the difference in energy of the sextet and quartet ground states in the relaxed Fe-N scan. (c) The weights of leading quartet CSFs defined in Eq. 2, $^4\Psi_1$ (solid blue), $^4\Psi_2$ (dot-dashed blue), $^4\Psi_3$ (dashed blue) and $^4\Psi_5$ (dotted blue) are shown. The weights of the two leading sextet CSFs defined in Eq. 3, $^6\Psi_1$ (solid red) and $^6\Psi_2$ (dashed red) are shown.

S3.1 Charge models on rigid scan

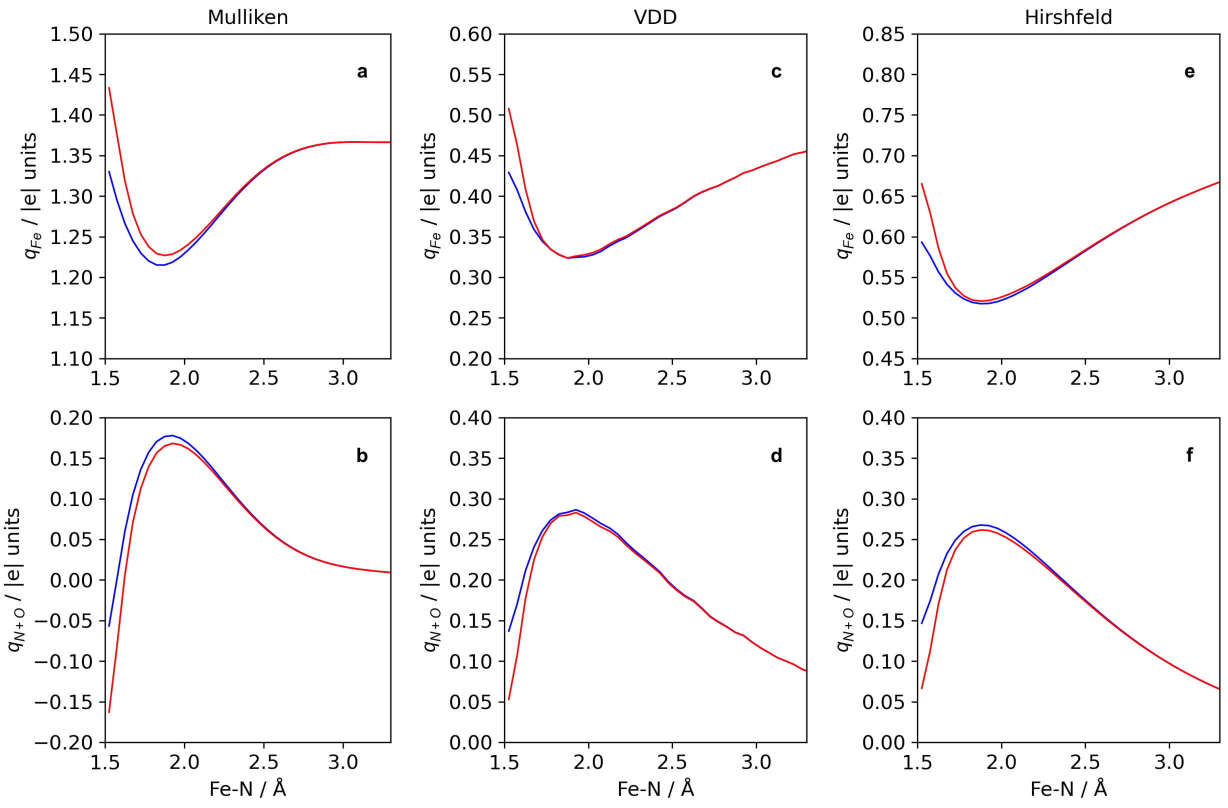

Figure S14: Different charge models along the rigid Fe-N scan for the q_{Fe} and q_{N+O} atoms and fragments, respectively. (a/b) The Mulliken atomic charges. (c/d) The Voronoi deformation density (VDD) atomic charges. (e/f) The Hirschfeld atomic charges.

S4 Configurational sampling

S4.1 Fe-N relaxed scan sampling

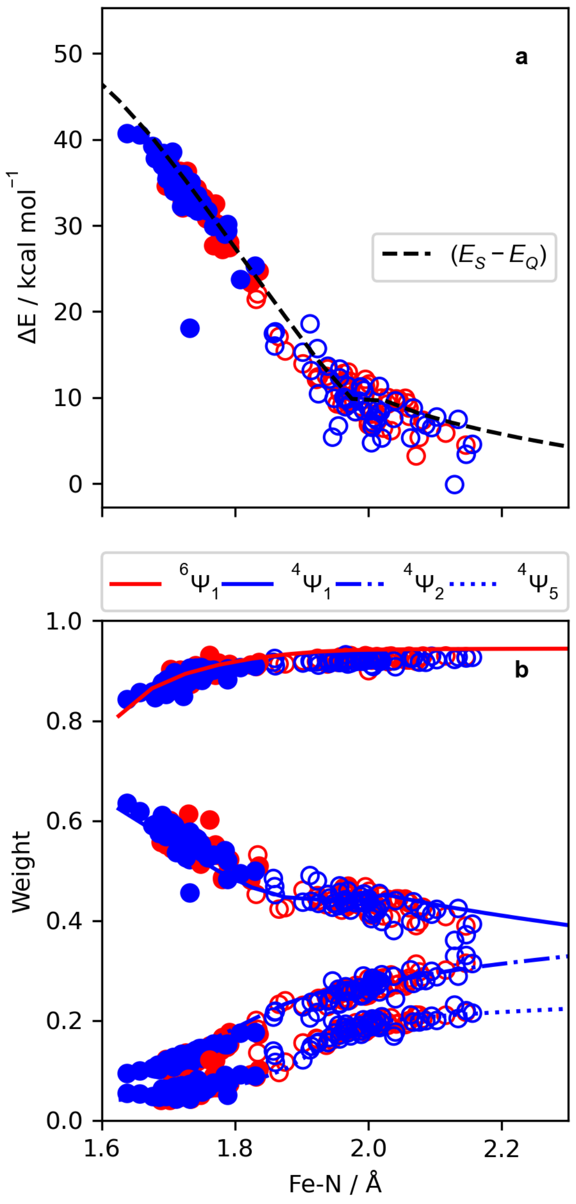

Figure S15: Comparison to Fig. 8 but using the relaxed scan along Fe-N distances (1.52 Å to 3.02 Å) optimized at the SS(1Q)-CAS(13e,10o)/def2-TZVP+cpcm(water) level of theory.

S4.2 Charge models

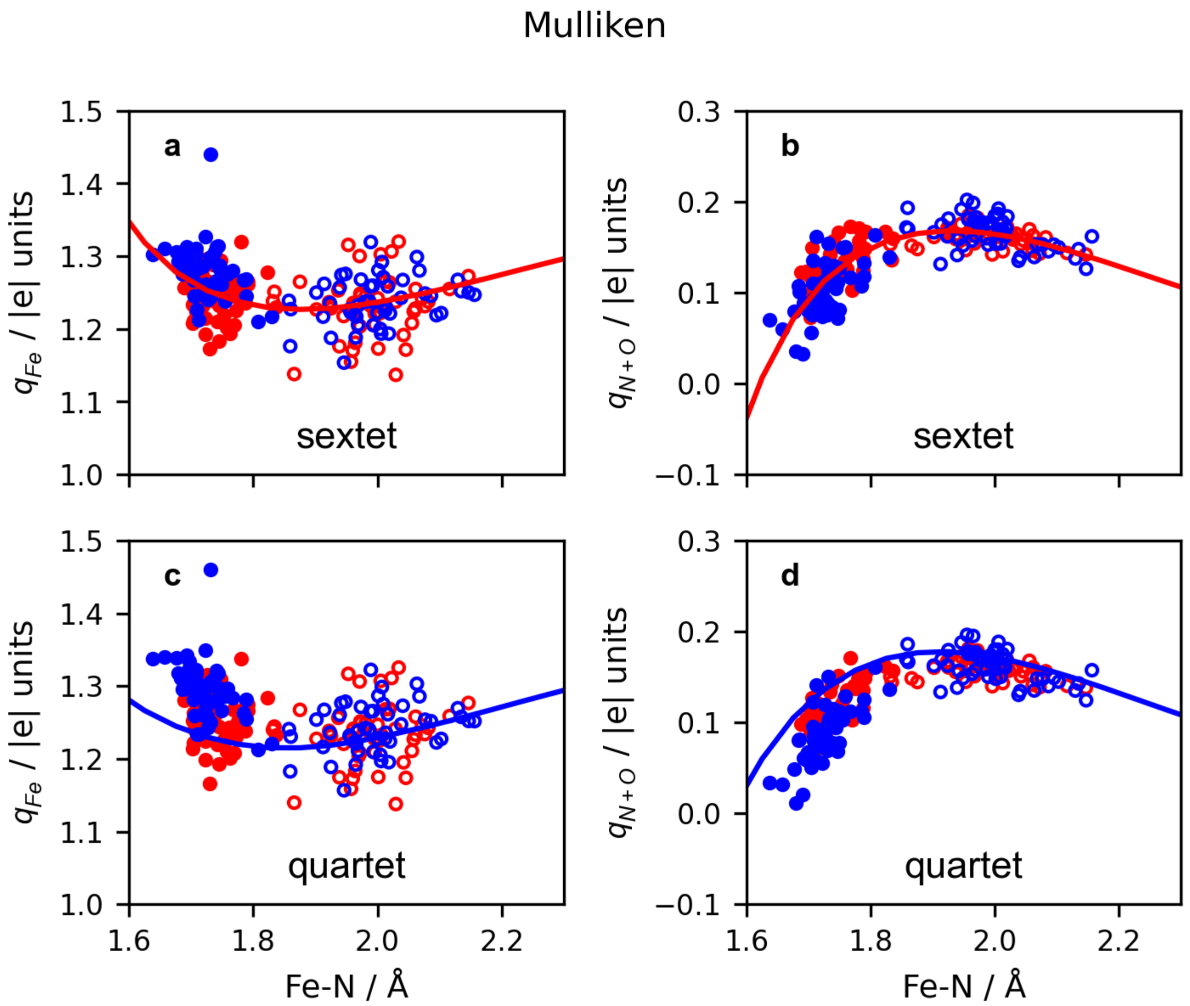

Figure S16: The Mulliken atomic charges along the rigid Fe-N scan for the q_{Fe} and q_{N+O} atoms and fragments, respectively. The atomic spin densities obtained from the sextet calculations (top) and quartet calculations (bottom) are shown. The atomic charges from the configuration sampling are shown according to the colouring scheme in Fig. 8(b).

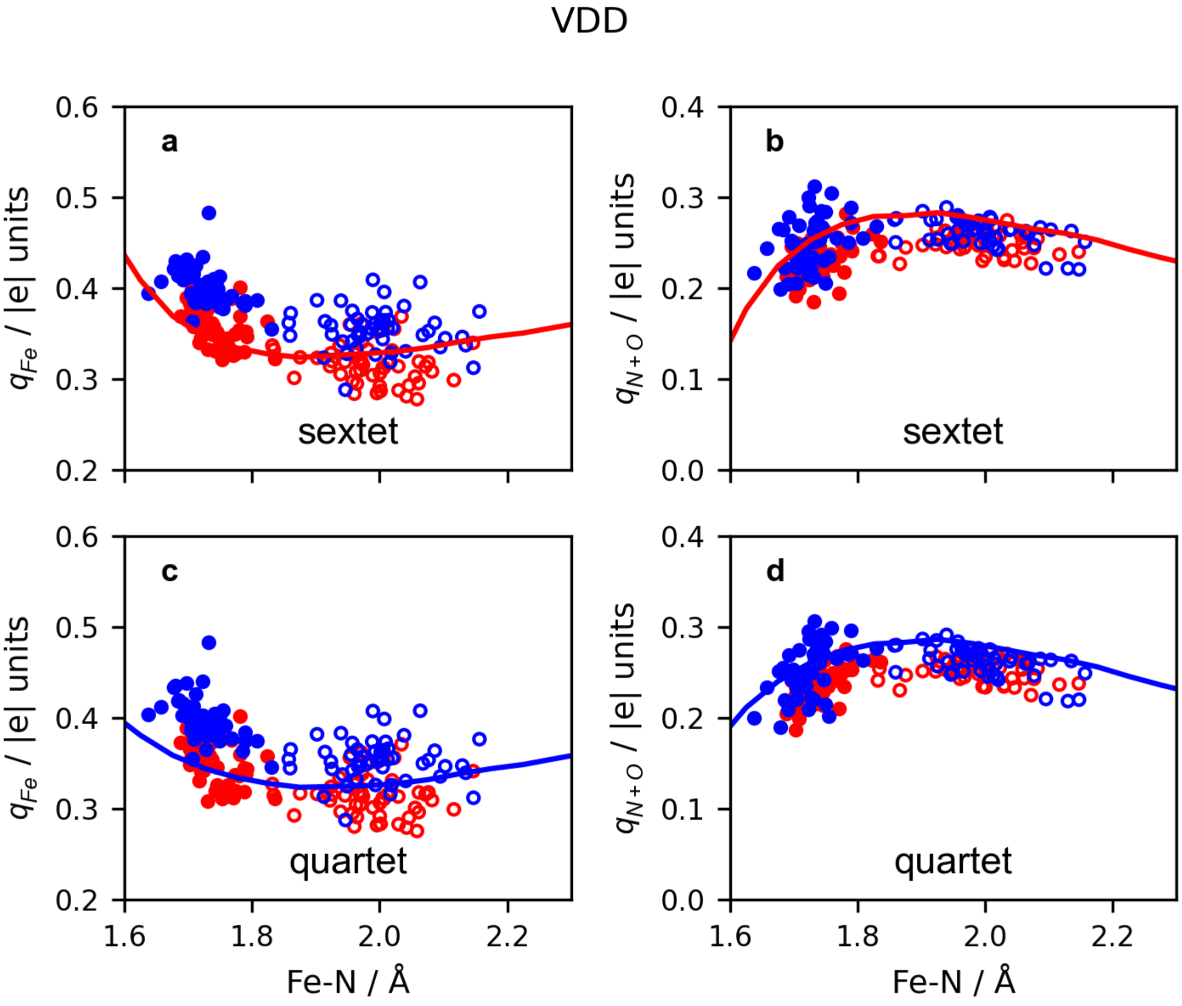

Figure S17: The Voronoi deformation density (VDD) atomic charges along the rigid Fe-N scan for the q_{Fe} and $q_{\mathrm{N+O}}$ atoms and fragments, respectively. The atomic spin densities obtained from the sextet calculations (top) and quartet calculations (bottom) are shown. The atomic charges from the configuration sampling are shown according to the colouring scheme in Fig. 8(b).

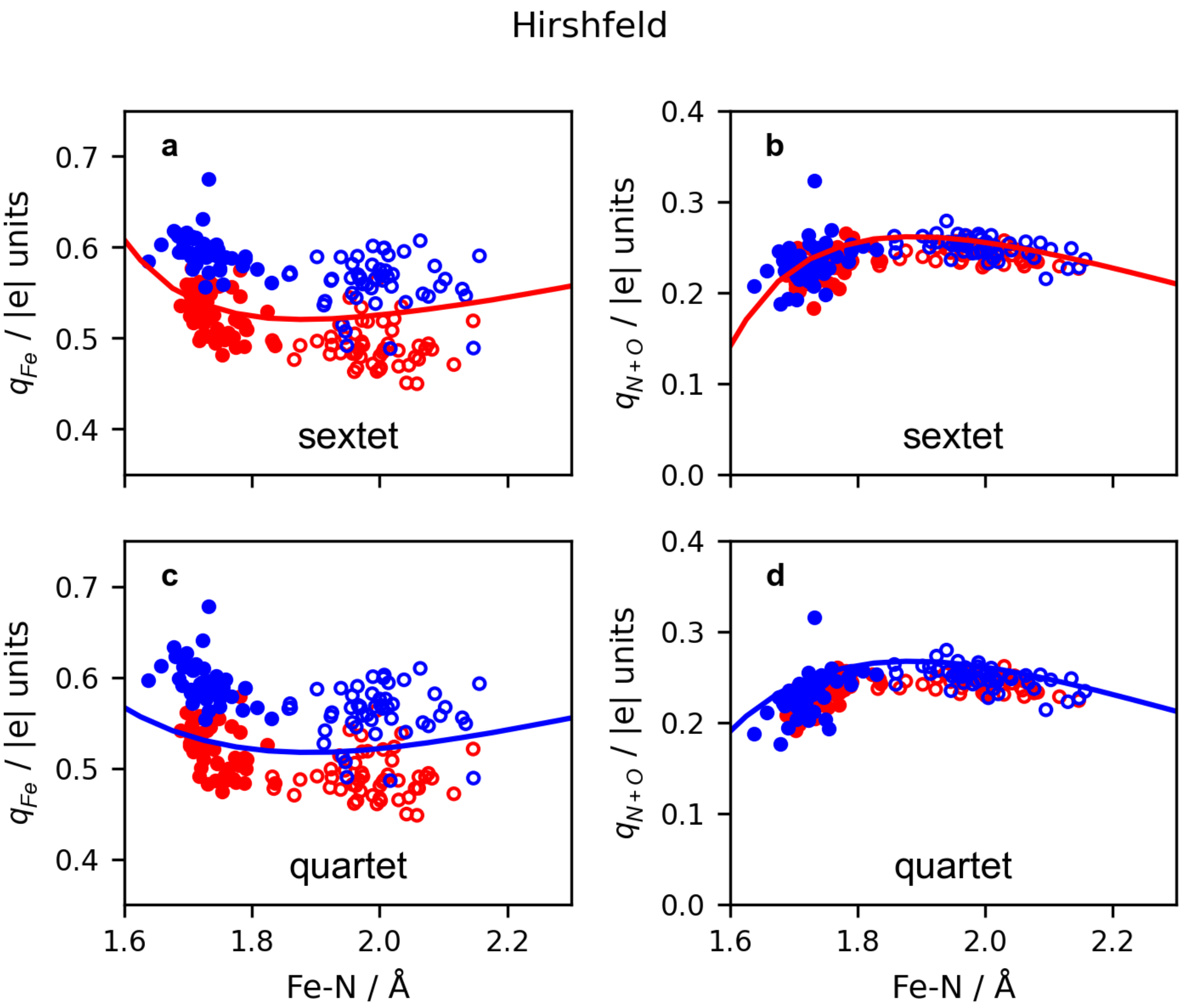

Figure S18: The Hirshfeld atomic charges along the rigid Fe-N scan for the q_{Fe} and q_{N+O} atoms and fragments, respectively. The atomic spin densities obtained from the sextet calculations (top) and quartet calculations (bottom) are shown. The atomic charges from the configuration sampling are shown according to the colouring scheme in Fig. 8(b).

S5 Analysis based on the CASSCF wavefunction

Table S5: The active space orbital ordering along the Fe-N reaction coordinate. The d_{xz} orbital that is changing between each geometry is bolded. At an Fe-N asymptotic distance of 3.32 Å, the π_y and d_{yz} are bolded to denote the shift of orbital ordering.

Fe-N / Å	Active space orbitals
1.72	$(\sigma)(\pi_y)(\pi_x)(d_{xz})(d_{yz})(d_{z^2})(d_{xy})(d_{x^2-y^2})(\pi_y^*)(\pi_x^*)$
1.92	$(\sigma)(\pi_y)(\mathbf{d_{xz}})(\pi_x)(d_{yz})(d_{z^2})(d_{xy})(d_{x^2-y^2})(\pi_y^*)(\pi_x^*)$
2.12	$(\sigma)(\mathbf{d_{xz}})(\pi_x)(\pi_y)(\pi_x)(d_{yz})(d_{z^2})(d_{xy})(d_{x^2-y^2})(\pi_y^*)(\pi_x^*)$
2.32	$(\mathbf{d_{xz}})(\pi_x)(\sigma)(\pi_y)(\pi_x)(d_{yz})(d_{z^2})(d_{xy})(d_{x^2-y^2})(\pi_y^*)(\pi_x^*)$
3.32	$(d_{xz})(\pi_x)(\sigma)(\pi_y)(\pi_x)(\boldsymbol{\pi_y^*})(d_{z^2})(d_{xy})(d_{x^2-y^2})(\mathbf{d_{yz}})(\pi_x^*)$

Table S6: The Mulliken analysis for $[Fe(H_2O)_5(NO)]^{2+}$ with an Fe-N distance of 5.5 Å and the associated fragments of $[Fe(H_2O)_5]^{2+}$ and NO. The CAS(13e,10o) active space is used for $[Fe(H_2O)_5(NO)]^{2+}$, the CAS(6e,5o) active space is used for $[Fe(H_2O)_5]^{2+}$ and the CAS(7e,5o) active space is used for NO. The CASSCF energies are reported in units of hartree.

	$[Fe(H_2O)_5(NO)]^{2+}$	$[Fe(H_2O)_5]^{2+}$	NO
E (CASSCF)	-1772.017529038	-1642.655551502	-129.3619378
σ_{Fe}	3.556445	3.951675	———
σ_{N+O}	-0.599979	———	1.00000
q_{Fe}	1.365133	1.365609	———
q_{N+O}	0.000484	———	0.00000

References

(1) Banerjee, A.; Coates, M. R.; Odelius, M. Spectroscopic Signature of Dynamical Instability of the Aqueous Complex in the Brown-Ring Nitrate Test. *Chem. Eur. J.* **2022**, *28*, e202200923.

(2) Neese, F.; Wennmohs, F.; Becker, U.; Riplinger, C. The ORCA quantum chemistry program package. *J. Chem. Phys.* **2020**, *152*, 224108.

(3) Radoń, M.; Broclawik, E.; Pierloot, K. Electronic Structure of Selected $\{FeNO\}^7$ Complexes in Heme and Non-Heme Architectures: A Density Functional and Multireference ab Initio Study. *J. Phys. Chem. B* **2010**, *114*, 1518–1528.

(4) Conradie, J.; Hopmann, K. H.; Ghosh, A. Understanding the Unusually Straight: A Search For MO Insights into Linear $\{FeNO\}^7$ Units. *J. Phys. Chem. B* **2010**, *114*, 8517–8524.

(5) Conradie, J.; Quarless, D. A.; Hsu, H.-F.; Harrop, T. C.; Lippard, S. J.; Koch, S. A.; Ghosh, A. Electronic Structure and FeNO Conformation of Nonheme Iron-Thiolate-NO Complexes: An Experimental and DFT Study. *J. Am. Chem. Soc.* **2007**, *129*, 10446–10456.
